# Artificial Intelligence in Arthroplasty: A Comprehensive Structured Critical Review and Descriptive Evidence Map of Validation, Uncertainty, and Workflow Integration

**DOI:** 10.3390/bioengineering13091036

**Published:** 2026-09-06

**Authors:** Furkan Yapıcı

**Affiliations:** Department of Orthopedics and Traumatology, Faculty of Medicine, Erzincan Binali Yıldırım University, 24100 Erzincan, Türkiye; furkanyapici@hotmail.com

**Keywords:** artificial intelligence, arthroplasty, clinical decision support, machine learning, implant recognition, natural language processing, human-in-the-loop, workflow optimization, periprosthetic joint infection, precision support

## Abstract

**Background/Objectives:** Artificial intelligence (AI) in arthroplasty spans clinical prediction, imaging, implant identification, planning, natural language processing, infection support, patient communication, and workflow optimization, but its translational readiness remains uncertain. This review evaluated how often current studies move beyond internal model development toward credible external evaluation, uncertainty-aware reporting, and clinically meaningful integration. **Methods:** A structured critical narrative review and descriptive evidence map included 252 publications identified and verified between 1 January and 17 August 2026. Of these, 172 were core arthroplasty-specific primary-AI publications used for task mapping, whereas 150 involved an assessable fitted model or operational system suitable for transportability analysis; 139 publications belonged to both analytical sets. Transportability was assessed separately based on geographic/site independence, temporal separation, and model state at the time of evaluation. **Results:** Strict frozen-model external evaluation was identified in 27/150 publications (18.0%). Twelve of these 27 publications (44.4%) belonged to a single commercial planning program; treating those 12 publications as a single program-level evidence unit reduced the count from 27 to 16. Later, non-overlapping temporal evaluation was established in 7/150 publications (4.7%), while the temporal relationship remained unclear in 86/150 (57.3%). In eight of the 150 transport-assessable publications (5.3%), external-validation terminology mapped to a different operational category under the framework used here. Among the 172 core publications, confirmed-present lower bounds were 27/172 (15.7%) for calibration, 5/172 (2.9%) for uncertainty or out-of-distribution handling, and 8/172 (4.7%) for observed workflow or patient benefit. The strongest near-term evidence concerned bounded, auditable tasks such as implant recognition, measurement, planning, and structured information extraction. **Conclusions:** Arthroplasty AI is best positioned as clinician-supervised precision-support infrastructure. Credible translation requires clearly defined evaluation settings, transparent reporting of model state, calibration and uncertainty assessment where applicable, and prospective workflow-level evaluation.

## 1. Introduction

Every arthroplasty procedure leaves behind a computational footprint. Before surgery, it includes comorbidity profiles, preoperative radiographs, CT or MRI data, templating decisions, laboratory values, patient-reported outcome measures, and expectations about recovery. During surgery, it expands to implant selection, fixation strategy, alignment targets, operative time, intraoperative findings, and technical workflow. After surgery, it continues through postoperative radiographs, infection surveillance, revision risk monitoring, registry capture, patient communication, and long-term functional outcomes. These data are not merely administrative by-products of surgical care; they are the raw material from which artificial intelligence (AI) systems may support the next generation of arthroplasty practice. Broad reviews already map this footprint across clinical prediction, imaging, implant analysis, templating, NLP-enabled data infrastructure, and emerging patient-facing communication tools [1,2,3,4,5].

Yet the most important question is not whether AI will replace the arthroplasty surgeon. That framing is both clinically unrealistic and technically misleading: arthroplasty is not a single prediction problem, and surgical judgment cannot be reduced to a model output. The more relevant question is whether AI can be engineered into the arthroplasty system so that surgical care becomes more measurable, reproducible, and efficient while clinician accountability is preserved. The future of arthroplasty AI, in other words, is less about autonomous decision-making than about clinician-supervised precision support—an emphasis that recurs in methodological critiques and in broader orthopedic AI syntheses, which tie meaningful adoption to clinical relevance, external validation, transparency, governance, and workflow-aware implementation rather than to algorithmic novelty or autonomous replacement [2,3,6,7,8].

AI in arthroplasty already spans a wide range of computational methods and clinical tasks. Supervised machine learning has predicted complications, readmission, mortality, discharge destination, length of stay, patient-reported improvement, satisfaction, and revision risk. Deep learning and computer vision have been applied to radiographic classification, implant recognition, automated component measurement, loosening detection, and postoperative imaging surveillance. Natural language processing (NLP) extracts structured arthroplasty variables from operative notes, radiology reports, and other free-text records. Large language models (LLMs) and chatbots have entered the field for patient education, perioperative communication, informed consent support, and multilingual engagement. Robotics-linked planning, CT segmentation, three-dimensional templating, and workflow-optimization studies show, in addition, that AI is becoming an enabling layer across the whole arthroplasty pathway rather than a narrow add-on to risk prediction [1,9,10,11,12,13,14,15,16].

The evidence, however, is uneven. Reported performance is usually strongest when the task is bounded, technically constrained, and directly verifiable—implant identification, automated cup-angle measurement, femoral stem subsidence quantification, component sizing, radiographic classification, and operative-note abstraction. In these settings, model outputs can be checked against clear labels, measurable geometry, or structured documentation. Broader prognostic models for readmission, satisfaction, patient-reported improvement, revision, and long-term outcomes are more heterogeneous, because their outcomes depend not on surgical technique and implant factors alone but also on baseline symptoms, mental health, expectations, comorbidity burden, social determinants, rehabilitation, and health system behavior. Technically impressive performance therefore does not automatically translate into clinical usefulness—a gradient evident in the literature itself, where high-performing bounded imaging and NLP studies sit alongside far more variable clinical prediction and PROM models [7,17,18,19,20,21,22,23,24].

This distinction matters for translation. The value of arthroplasty AI cannot be judged by discrimination metrics alone: a high area under the curve does not show that a model improves counseling, changes treatment, reduces complications, saves time, or improves operating room efficiency, just as a fluent chatbot response is not necessarily safe, complete, or appropriate for unsupervised patient guidance. To become clinically credible, a model must be externally validated, calibrated, interpretable where possible, uncertainty aware, resistant to dataset shift, and compatible with real clinical workflow. The core engineering challenge is therefore system integration, not model development alone—a point made repeatedly in arthroplasty AI reviews and methodological critiques, and most visible in studies that compare machine-learning models against external validation, conventional baselines, or real-world communication constraints [2,3,6,7,10,13,14,24].

Seen this way, arthroplasty AI is a layered clinical engineering infrastructure. The input layer holds radiographs, CT, MRI, laboratory values, patient-reported outcomes, operative notes, implant metadata, registries, administrative data, and patient-generated information. The analytic layer holds machine learning, deep learning, computer vision, NLP, LLMs, radiomics, segmentation, optimization, and hybrid multimodal systems. The task layer covers risk prediction, implant recognition, radiographic measurement, templating, sizing, infection support, registry construction, patient communication, robotics-linked planning, and workflow optimization. The validation layer covers external testing, calibration, uncertainty quantification, comparator reporting, explainability, and decision curve or workflow-impact analysis. The deployment layer covers surgeon-facing dashboards, PACS integration, registry pipelines, operating room scheduling tools, robotic planning systems, and supervised patient-facing communication platforms. The framework is drawn from the arthroplasty AI domains now represented in the literature, including implant analysis, three-dimensional templating, NLP-enabled registry construction, PJI modeling, patient-facing LLM studies, and uncertainty-aware implant-identification systems [10,11,12,14,19,25,26,27,28].

Earlier reviews have summarized AI in total joint, hip, knee, and shoulder arthroplasty, in implant analysis, NLP, three-dimensional templating, foot-and-ankle surgery, and PJI-focused decision support. Many are valuable but remain organized by joint, algorithm family, or clinical endpoint—including the most recent broad orthopedic AI reviews. One offers a broad synthesis of AI across orthopedic bone care, spanning biomaterials, smart implants, bone regeneration, and neuroprosthetics [29]; another, restricted to original studies and organized by anatomical region across hip, knee, shoulder, and spine, concentrates on implant positioning, dislocation risk prediction, and surgical indications [30]. A recent structured narrative review addressed orthopedic AI more broadly, from diagnosis to follow-up, around the changing role of the surgeon [8]. What is still missing is a structured critical synthesis that treats arthroplasty AI as an integrated system—one linking imaging, implants, text, prediction, infection, planning, robotics, communication, validation, and workflow. Such a synthesis is timely because the field has moved past proof-of-concept prediction toward a harder translational question: which AI tools are mature enough for clinician-supervised deployment, and which remain limited by validation, generalizability, explainability, or workflow barriers [1,4,5,9,10,11,12,31,32,33,34,35]? The present review differs from these works in three respects: it is confined to arthroplasty rather than orthopedics as a whole; it is organized by translational engineering function rather than by anatomical region or device category; and it pairs a reference-level evidence map with a domain-by-domain appraisal of how close each application has come to safe, surgeon-supervised deployment. The NLP-based data-infrastructure, periprosthetic infection, large language model, and workflow domains synthesized here are, in particular, not covered by either recent review [29,30].

This review therefore examines AI in arthroplasty from a clinical engineering and translational systems perspective, with five objectives: to synthesize the major application domains of AI across arthroplasty care; to separate bounded, verifiable tasks that have clearer near-term workflow pathways from broader prognostic models of less certain clinical utility; to evaluate the roles of imaging automation, implant recognition, templating, NLP, PJI support, LLMs, robotics-linked planning, and workflow optimization; to identify recurring validation, explainability, calibration, and implementation gaps; and to propose an engineering-oriented framework for safe, human-in-the-loop arthroplasty AI. Its central argument is that AI’s most credible future in arthroplasty lies in system engineering, not surgeon replacement: that is, precision support that works with imaging and implant data, fits clinical workflows, and strengthens surgical judgment while accountability stays clinician-led.

## 2. Materials and Methods

### 2.1. Review Design, Evidence Mapping, and Conceptual Framework

The article was designed as a structured critical review supported by transparent literature identification, reference-level evidence mapping, and a descriptive evidence profile reporting six design and reporting columns per task category. It was not intended to function as a prospectively registered systematic review, meta-analysis, scoping review, or formal certainty-of-evidence assessment. Instead, its purpose was to synthesize how artificial intelligence (AI) is being engineered across the arthroplasty pathway from a clinical engineering and translational systems perspective, with particular attention to imaging automation, implant recognition, templating and sizing, natural language processing (NLP), periprosthetic joint infection (PJI) support, large language models (LLMs), robotics-linked planning, and workflow optimization.

Rationale for review design. A structured critical narrative design was chosen because the purpose of this article was not to answer a narrowly framed intervention-effectiveness question, estimate pooled diagnostic or prognostic accuracy, or map all available evidence according to a formal scoping review protocol. Instead, the objective was to synthesize how AI is being engineered across heterogeneous arthroplasty tasks, including imaging automation, implant recognition, templating, natural language processing, periprosthetic joint infection support, large language models, robotics-linked planning, and workflow optimization. The evidence map was therefore used to improve transparency, traceability, and domain-level synthesis, but it should not be interpreted as an exhaustive systematic evidence base or as a formal certainty-of-evidence framework.

The review was organized around the central premise that arthroplasty AI should be evaluated not as a collection of isolated prediction models, but as a developing precision-support infrastructure. Accordingly, evidence was interpreted across five interrelated layers: data inputs, including imaging, implant metadata, operative notes, registries, laboratory values, and patient-reported outcomes; analytic methods, including machine learning, deep learning, computer vision, NLP, LLMs, radiomics, segmentation, and optimization; clinical engineering tasks, including prediction, planning, recognition, measurement, documentation, communication, and workflow support; validation maturity, including external validation, calibration, comparator reporting, explainability, and uncertainty handling; and deployment relevance, including compatibility with surgeon-facing, registry-facing, patient-facing, and workflow-facing clinical systems.

The Supplementary Materials are organized as follows. Supplementary Table S1A provides the 252-publication reference map. Supplementary Table S1B contains aligned publication-level sheets covering evidence role, source depth, applicability routing, validation design, transportability, terminology-to-design mapping, and confirmed reporting and deployment signals. Supplementary Table S2 summarizes the literature identification and source verification framework and the analytical denominators. Supplementary Table S3 defines the descriptive evidence profile rules, and Supplementary Table S4 provides criterion-specific task-level numerators and denominators, sensitivity analyses, counting rules, and detailed publication allocation.

Formal scoping review guidance was consulted as a reporting reference, but the article does not claim PRISMA-ScR compliance because it was not designed as a protocol-driven scoping review [36].

### 2.2. Review Objectives

The objectives of this structured critical narrative review were:

1. To synthesize the major domains in which AI has been applied across arthroplasty care.
2. To distinguish bounded and verifiable AI tasks with clearer near-term workflow pathways from broader prognostic models with less certain clinical utility.
3. To evaluate the translational roles of imaging automation, implant recognition, templating, NLP, PJI support, LLMs, robotics-linked planning, and workflow optimization.
4. To identify recurring validation, explainability, calibration, and implementation gaps.
5. To propose a clinical engineering framework for safe, human-in-the-loop arthroplasty AI.

### 2.3. Literature Identification Strategy

Literature identification (database searching, citation tracking, and manual record retrieval), source acquisition, and source verification were conducted between 1 January and 17 August 2026. PubMed/MEDLINE, Web of Science Core Collection, Scopus, and Google Scholar were used to identify relevant literature on AI in arthroplasty. No lower publication year restriction was applied. The substantive arthroplasty AI literature in the evidence map dated from 2018 to 2026; three methodological framework references predated 2018.

Search terms combined arthroplasty-related concepts with AI-related concepts and were adapted to each database. Arthroplasty terms included total joint, hip, knee, shoulder, ankle, elbow, and thumb carpometacarpal arthroplasty. AI terms included artificial intelligence, machine learning, deep learning, neural network, computer vision, radiomics, natural language processing, large language model, and ChatGPT.

Database searching, backward and forward citation checking, and manual retrieval of clinically relevant or sparsely represented publications were integrated within the same literature identification process. This approach was used because the literature is distributed across orthopedic, radiology, biomedical-engineering, informatics, and digital-health journals. Google Scholar supported identification of difficult-to-locate publications and citation checking rather than functioning as a fully reproducible Boolean database. Supplementary Table S2 summarizes the search sources, literature identification period, source verification framework, analytical set routing, and manuscript representation.

**Figure 1:**
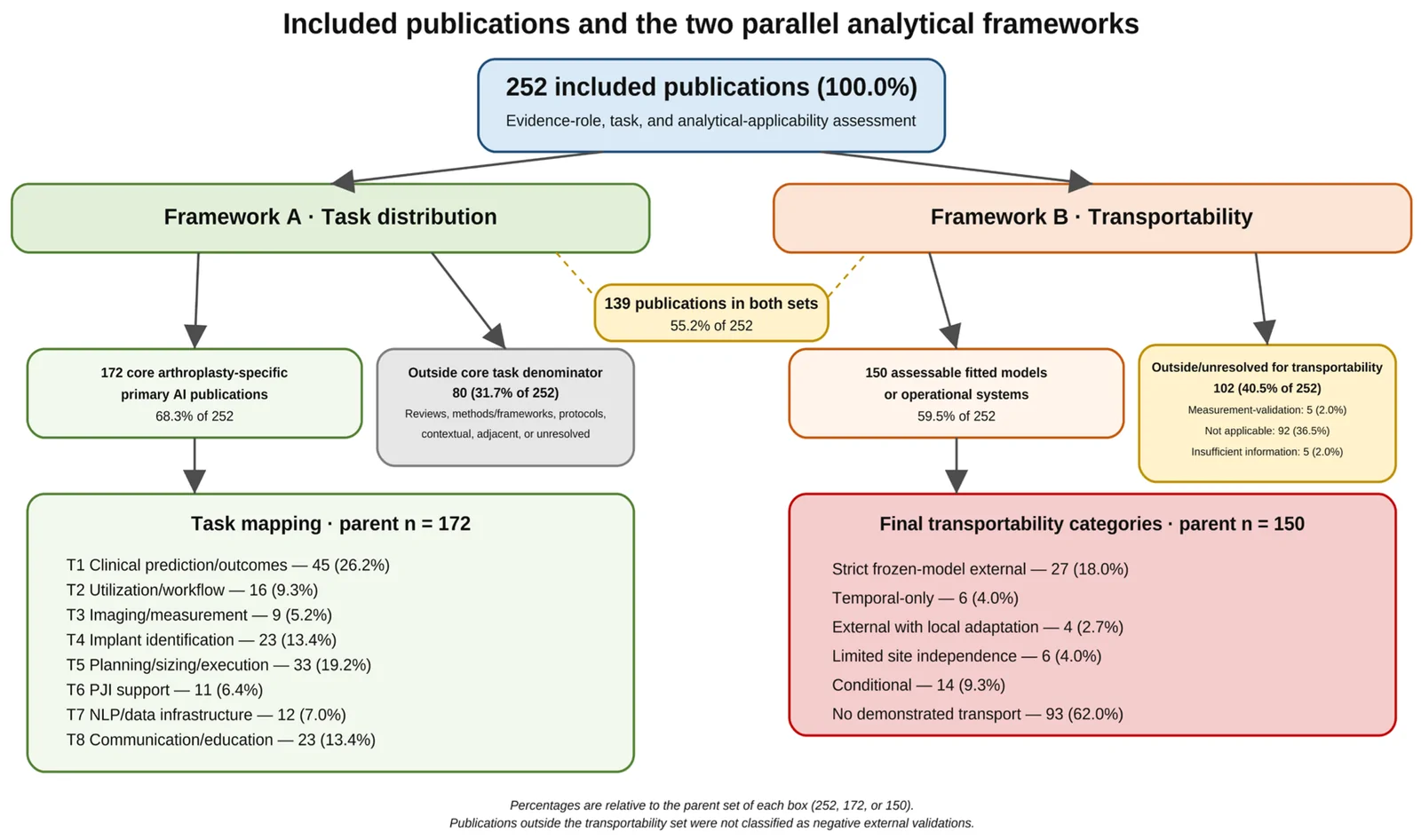
Included publications and the two parallel analytical frameworks. Percentages are relative to the parent set indicated in each box (252, 172, or 150). Of 252 included publications (100.0%), 172 (68.3%) formed the core task distribution set and 150 (59.5%) were transport-assessable; 139 publications (55.2%) belonged to both sets. The 172-publication task set was distributed across T1–T8, while 80 publications (31.7%) were outside that denominator. For transportability, 5 publications (2.0%) followed a measurement validation route, 92 (36.5%) did not present an applicable transport question, and 5 (2.0%) lacked sufficient information. The 150 transport-assessable publications were classified as strict frozen-model external (27; 18.0%), temporal-only (6; 4.0%), external with local adaptation (4; 2.7%), limited site independence (6; 4.0%), conditional (14; 9.3%), or no demonstrated transport (93; 62.0%). Publications outside the transportability set were not treated as negative external validations.

Figure 1 shows how the 252 included publications entered two parallel analytical frameworks and displays every count with a percentage relative to the parent set of the corresponding box. The first framework describes the distribution of 172 core arthroplasty-specific primary-AI publications across the T1–T8 task families. The second addresses model transportability in 150 publications for which a fitted model or operational system could be assessed. These frameworks overlap in 139 publications but answer different questions and should not be interpreted as sequential screening stages.

### 2.4. Source Selection Framework

Sources were selected if they contributed substantively to understanding AI applications in arthroplasty care, planning, imaging, implant analysis, infection support, data infrastructure, patient communication, robotics-linked planning, or workflow optimization. Eligible sources included original empirical studies, validation studies, registry and data-infrastructure studies, systematic reviews, scoping reviews, structured narrative reviews, methodological critiques, and patient-facing AI studies.

The population of interest included patients undergoing, being evaluated for, or being followed after arthroplasty procedures, as well as arthroplasty-related operative notes, radiographs, registries, implant datasets, perioperative records, and workflow systems. The concept of interest was the application of AI or AI-adjacent computational methods, including supervised machine learning, unsupervised learning, deep learning, computer vision, NLP, radiomics-based modeling, LLMs, chatbots, AI-assisted templating, implant recognition, AI-enabled registry extraction, and optimization-based workflow tools. The clinical context included preoperative planning, patient selection, implant sizing, digital templating, radiographic surveillance, implant identification, complication prediction, revision prediction, PJI support, registry construction, perioperative workflow optimization, patient education, and clinician-supervised communication.

Hip, knee, shoulder, ankle, elbow, and hand/thumb carpometacarpal arthroplasty studies were retained, along with general total joint arthroplasty studies. Temporomandibular joint studies were retained only when they were relevant to adjacent joint reconstruction AI themes and were interpreted separately from the core arthroplasty evidence. Editorials without substantive evidence contribution, non-human studies, non-arthroplasty orthopedic AI studies, conference abstracts without full text, duplicate records, and papers without sufficient arthroplasty-specific or AI-specific detail were excluded.

The analytic framework used to select, classify, and interpret the supporting literature is summarized in Table 1. The framework defines the population and clinical context, AI concept, clinical engineering task, data modality, AI family, evidence type, validation maturity, translational relevance, and deployment concerns used during evidence abstraction and narrative synthesis.

In this review, AI was used as an umbrella term for machine learning, deep learning, computer vision, NLP, LLM-based systems, radiomics-based modeling, and hybrid computational decision-support approaches. Conventional regression-only studies were not classified as AI unless they were explicitly embedded within an AI/ML comparative framework, used as a baseline comparator for ML models, or formed part of an AI-adjacent data-infrastructure or hybrid modeling pipeline.

Screening was performed in sequential stages. First, titles and abstracts were screened for arthroplasty relevance and the presence of an AI, machine-learning, deep-learning, NLP, LLM, radiomics, or computational workflow component. Second, potentially eligible full texts were reviewed to confirm arthroplasty specificity, task relevance, data modality, and sufficient methodological detail for reference-level mapping. Third, retained sources were assigned to a dominant clinical engineering task and an arthroplasty domain for evidence mapping. Exclusions at the full-text stage were based on lack of arthroplasty specificity, absence of an AI/ML/NLP/LLM component, insufficient full-text methodological detail, non-human or purely technical scope without clinical arthroplasty relevance, or non-English full text.

### 2.5. Included Evidence Set and Parallel Analytical Frameworks

The review included 252 publications organized at the reference level. Full-text or authoritative source files were read for 247 publications; five additional abstract-only contextual records were retained and were not used in primary quantitative results. Review articles, methodological and reporting papers, protocols, contextual publications, and empirical AI studies were retained for different narrative purposes rather than being treated as equivalent evidence units.

**Figure 2:**
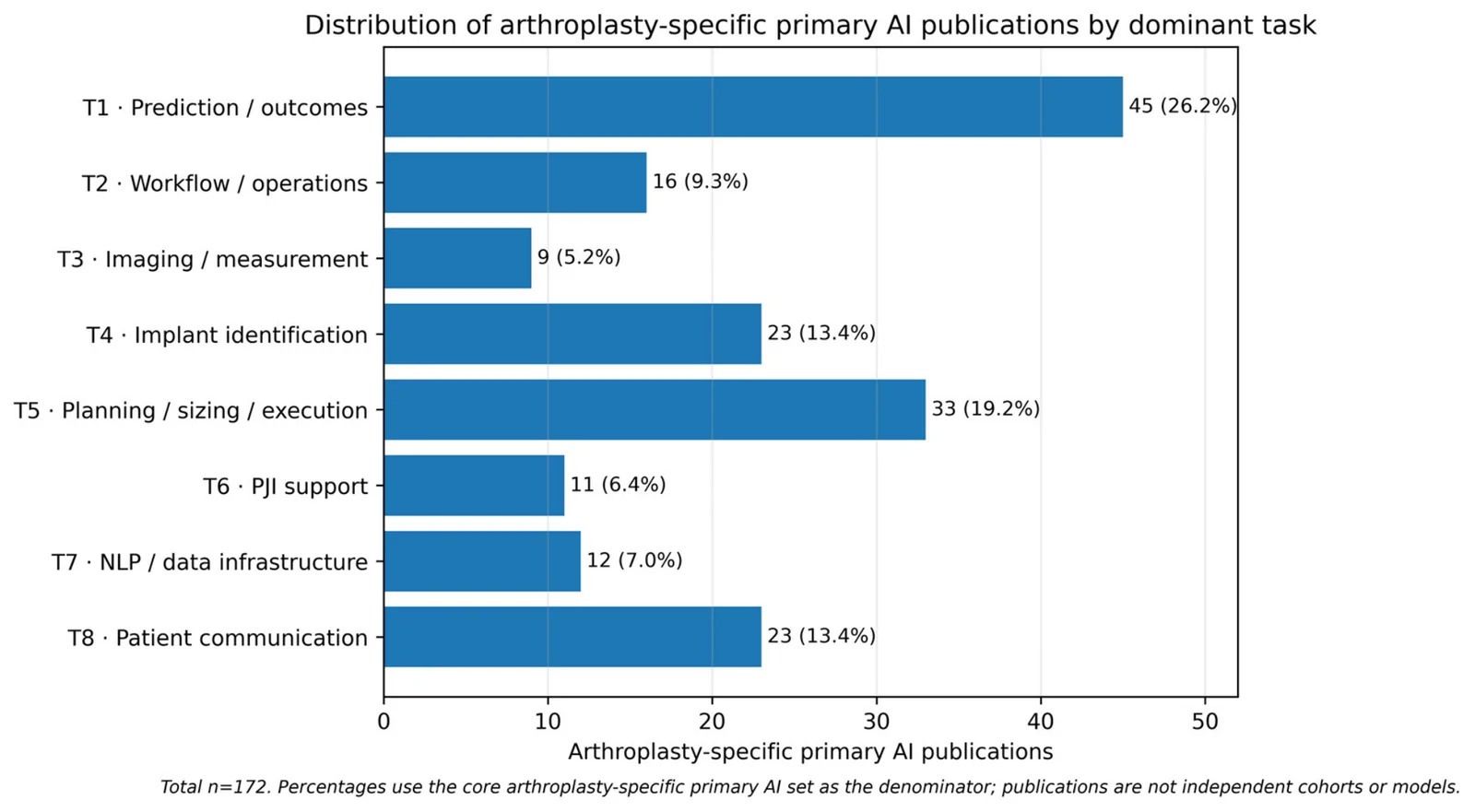
Distribution of arthroplasty-specific primary-AI publications by dominant task. The 172 core publications were allocated to T1 clinical prediction and outcomes (45; 26.2%), T2 utilization/workflow (16; 9.3%), T3 imaging/measurement/surveillance (9; 5.2%), T4 implant identification (23; 13.4%), T5 planning/sizing/execution (33; 19.2%), T6 PJI support (11; 6.4%), T7 NLP/data infrastructure (12; 7.0%), and T8 patient/clinician communication (23; 13.4%). The denominator is 172, not the 150-publication transportability set. Counts are publication level and do not represent independent cohorts or models.

Two parallel analytical frameworks were applied. First, 172 publications met the definition of core arthroplasty-specific primary-AI research and formed the denominator for the T1–T8 task distribution in Figure 2. Second, model transportability could be assessed in 150 publications involving an assessable fitted model or operational system. The two sets overlapped in 139 publications but were not identical. Thirty-three core arthroplasty AI publications were outside the transportability analysis (28 because the transport question was not applicable and five because they followed a measurement validation route), while 11 contextual or adjacent publications outside the core task distribution set remained informative for the transportability framework.

Reviews, methodological and reporting papers, protocols, contextual publications without an applicable transport question, measurement validation studies, and records without sufficient information were not counted as negative external validations. Consequently, task distribution percentages use 172 as the denominator, whereas model transportability percentages use 150. The 252-publication total describes the complete included evidence set and is not the denominator for every analytical question.

The unit of organization was the cited publication, not an independent patient cohort, model development dataset, or deduplicated clinical study. Publication-level counts therefore describe the mapped evidence set rather than independent datasets or non-overlapping populations. Review articles were used to contextualize domain-level trends, while original empirical studies were used to characterize model development, evaluation design, performance, and implementation relevance.

Supplementary Table S1A provides the compact publication-level map, and Supplementary Table S1B provides aligned publication-level fields for evidence role, source depth, task assignment, applicability routing, transportability, terminology-to-design mapping, and selected reporting and deployment signals. Supplementary Table S4 reports criterion-specific task denominators, sensitivity analyses, counting rules, and detailed publication allocation.

### 2.6. Evidence Abstraction and Classification

Each retained reference was assessed using a structured reference-level framework. The working extraction considered author, year, title, journal, arthroplasty domain, evidence category, study design, sample size where applicable, data source, AI method, algorithm family, input modality, target task or outcome, validation approach, reported performance metrics, comparator or calibration reporting, explainability or uncertainty features, workflow relevance, use in synthesis, and major limitations. The public Supplementary Table S1A,B report the publication-level fields used for the evidence map and descriptive analyses.

Every source available at sufficient depth was verified against the primary or authoritative record. Five records available only as abstracts were retained as a separate source-depth category and were not treated as evidence of absence. Detailed source verification notes and document locators were used during assessment but are not included in the public Supplementary Materials.

Sources were classified across four primary analytic axes:

1. **Arthroplasty domain**, including general total joint arthroplasty, hip, knee, shoulder, ankle, elbow, hand/thumb carpometacarpal arthroplasty, mixed arthroplasty populations, and adjacent TMJ evidence.
2. **Primary clinical engineering tasks**, including clinical prediction, utilization modeling, templating, sizing, planning, implant identification, radiographic measurement, postoperative surveillance, PJI support, NLP or registry extraction, patient education, LLM-based communication, robotics-linked segmentation, workflow optimization, and implant design engineering.
3. **AI family**, including supervised machine learning, unsupervised learning, deep learning, computer vision, NLP, LLM/chatbot systems, radiomics-based modeling, hybrid or multimodal AI, and optimization-based engineering tools.
4. **Validation and translational design**, including internal holdout or cross-validation, temporal evaluation, site-separated evaluation, independent geographic evaluation, local adaptation or redevelopment, qualitative application evaluation, and unresolved design.

When a source addressed more than one domain, one dominant synthesis task was assigned for primary allocation, while secondary anatomical or application relevance was retained as a cross-cutting field. Review-level evidence, methodological critiques, reporting guidelines, and contextual papers were retained for interpretation but were not treated as independent empirical model evaluations.

Validation design was classified from the full text using the operational definitions in Table 2. The terminology used in each publication was compared with the design established in the Methods section so that differences could be described without treating either as an error. A four-state applicability route distinguished transport-assessable studies, measurement validation studies, records for which transport questions were not applicable, and records with insufficient information; this route is reported for all 252 publications in Supplementary Table S1B. For the 150 transport-assessable publications, three core axes were recorded: geographic/site independence, later non-overlapping temporal separation, and model state at evaluation (frozen versus adapted, recalibrated, retrained, or redeveloped). Source silence was recorded as unclear, not as no.

A strict frozen-model geographic external class was derived only when the evaluation setting was geographically/site independent, and the evaluated model was applied without local retraining or adaptation. Same-site temporal evaluation, site-held-out evaluation inside a shared organization or data environment, local adaptation, model updating, independent data redevelopment, product/domain transport, and open-set testing were retained as distinct properties rather than collapsed into one binary label. Investigator, funding/program, reference standard, product/domain, and open-versus-closed-set independence were retained as additional qualifiers where source-supported. Examples of terminology and design mapping are presented neutrally in §4.4.

The operational categories, their definitions, and their treatment in the analysis are set out in Table 2.

### 2.7. Evidence Profile

Each task category was described using six evidence profile columns: independent geographic or site-separated evaluation; later non-overlapping temporal evaluation; multicenter or multi-source development data; performance completeness and decision utility; uncertainty or failure-mode handling; and observed workflow or patient benefit evidence.

Eligibility was defined before the reporting signal was assessed. For each column, eligibility was determined from study design without reference to whether the publication reported a favorable signal. Measurement and segmentation studies, for example, were not treated as eligible for probabilistic calibration criteria when they did not produce individual risk estimates. This design-first rule prevents the denominator from being shaped by the observed result.

**Figure 3:**
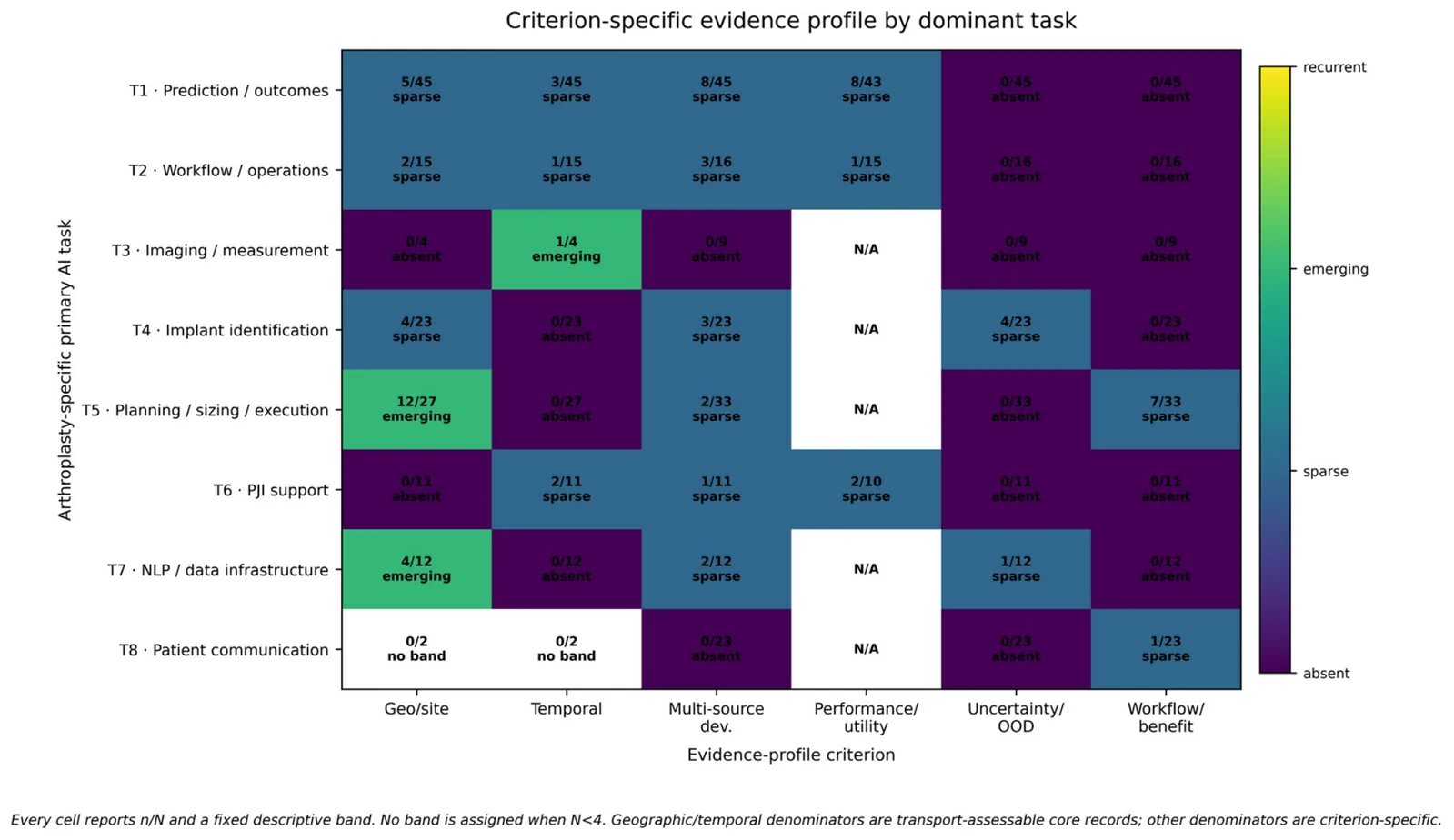
Criterion-specific evidence profile by dominant task. Each cell reports the confirmed positive signal and eligible denominator for geographic/site-separated evaluation, later non-overlapping temporal evaluation, multicenter or multi-source development, performance completeness and decision utility, uncertainty/OOD handling, and observed workflow or patient benefit. Bands are absent (0.0%), sparse (>0.0% to <25.0%), emerging (25.0% to <50.0%), and recurrent (≥50.0%); no band is assigned when the eligible denominator is below four. Geographic and temporal denominators use transport-assessable core publications. Multicenter, uncertainty, and workflow fields are confirmed-present lower bounds. The underlying *n*/*N* values are the primary results; the bands are descriptive visual aids only and should not be interpreted as validated evidence-certainty or maturity categories or as comparative-effectiveness rankings.

Reporting rule. Every Figure 3 cell shows the numerator, eligible denominator, and a descriptive band. The same values are tabulated in Supplementary Table S4 for detailed review. Bands were defined as absent (0.0%), sparse (>0.0% to <25.0%), emerging (25.0% to <50.0%), and recurrent (≥50.0%). When the eligible denominator was below four, the cell reports *n*/*N* without a band. The underlying numerator and eligible denominator *(n*/*N*) remain the primary results; the bands are descriptive visual aids and are not validated evidence-certainty, quality, or maturity categories.

Performance completeness required at least one calibration or overall-error measure together with at least one class-sensitive or decision-utility measure. Discrimination alone did not qualify. Uncertainty handling required a mechanism that could decline or flag an input; post hoc attribution methods alone did not qualify. Workflow evidence required an outcome measured in a real clinical or operational setting rather than a statement of possible integration or workstation processing time.

### 2.8. Critical Narrative Synthesis

Evidence synthesis was performed descriptively and thematically. First, the included evidence set was summarized by arthroplasty domain, primary task, AI family, data modality, and validation maturity. Second, a structured critical synthesis was conducted across the major translational domains represented in the literature: clinical prediction, implant recognition, imaging automation, templating and sizing, NLP-enabled data infrastructure, PJI support, LLM-based patient communication, robotics-linked planning, and workflow optimization.

Third, findings were interpreted through a clinical engineering systems framework. This framework emphasized the relationship between input data, model architecture, task definition, validation method, clinical interpretability, and deployment context. Particular attention was paid to the distinction between bounded and verifiable AI tasks, such as implant identification, automated radiographic measurement, component sizing, and operative-note abstraction, and broader prognostic tasks, such as readmission, satisfaction, patient-reported improvement, revision, or long-term outcome prediction.

No meta-analysis was attempted because the included literature was heterogeneous with respect to joint domain, data source, AI method, study design, validation strategy, target outcome, and reported performance metric. Performance estimates were therefore interpreted narratively and task-specifically rather than pooled across domains.

### 2.9. Methodological Boundaries of the Review

This article is a structured critical narrative review supported by a source-verified evidence map and descriptive quantitative summaries. It was designed to compare recurring validation, uncertainty, and workflow integration patterns across heterogeneous arthroplasty AI applications, not to estimate pooled treatment effects or produce formal certainty-of-evidence ratings. All quantitative results therefore describe the included evidence set and the stated eligibility denominator rather than field-wide prevalence.

Publications are not independent evidence units. When several publications shared a fitted model, development dataset, clinical site/window, or clearly documented program, the relationship was recorded and explored in a lineage sensitivity analysis. Shared authorship alone was not sufficient. The primary strict external evaluation count was publication-level (27). Both same-site/window publication pairs were contained within the 12-publication commercial planning program cluster. The two lineage analyses were therefore treated as alternative, non-cumulative sensitivity scenarios: collapsing the two same-site/window pairs reduced the count from 27 to 25, whereas separately treating all 12 program publications as one program-level evidence unit reduced the count directly from 27 to 16 (27 − 12 + 1). The program-level count was not calculated from 25.

Literature selection, structured extraction, classification, and synthesis were performed by the sole author. Full-text or authoritative source files were reviewed for 247 of the 252 included publications, while five abstract-only contextual records were retained; none contributed to primary quantitative results. Publication identity, task assignment, validation category, performance fields, and source notes were checked repeatedly against the source documents and the working evidence table. Independent dual screening and duplicate extraction were not available and remain limitations.

The evidence profile is descriptive rather than a formal risk-of-bias assessment. It reports six criterion-specific signals with explicit eligibility denominators. The values should not be interpreted as quality scores, evidence-certainty grades, omission prevalence, or comparative-effectiveness rankings.

Two sensitivity analyses were retained. First, lineage sensitivity compared the publication-level strict count (27) with two alternative, non-cumulative scenarios: the same-site/window-pair collapse (25) and the commercial-program-collapse upper bound (16). Second, source-depth sensitivity confirmed that the five abstract-only publications did not enter the 150-publication transportability denominator and did not alter any primary quantitative result.

### 2.10. Assessment Procedure and Single-Author Limitation

All structured extraction, classification, source verification, and final judgments were performed by the author. Duplicate independent assessment by a second assessor was not available. To improve transparency, the task definitions, publication-level assessment fields, applicability routes, terminology-to-design mapping, and standardized terminology used in the public evidence map are reported in Supplementary Table S1B, while eligibility and counting rules are reported in Supplementary Table S4. Detailed source locators were used during assessment but are not included in the public Supplementary Materials.

## 3. Critical Synthesis

### 3.1. Evidence Set Composition and Verification Status

The 252 included publications comprised 175 primary-AI method/application records (125 method, 48 application, and two method-and-application), 45 secondary reviews, 11 methods or appraisal frameworks, 10 primary-context publications without a documented AI method, three protocols, three adjacent non-arthroplasty primary-AI publications, and five abstract-only contextual records excluded from the 172-publication task and 150-publication transportability denominators. All 252 publications are listed in Supplementary Table S1A.

Of the 252 publications, 172 were core arthroplasty-specific primary-AI studies and formed the denominator for the task distribution shown in Figure 2. They were assigned to T1 clinical prediction (45; 26.2%), T2 utilization/workflow (16; 9.3%), T3 imaging/measurement (9; 5.2%), T4 implant identification (23; 13.4%), T5 planning/sizing/execution (33; 19.2%), T6 PJI support (11; 6.4%), T7 NLP/data infrastructure (12; 7.0%), and T8 communication/education/consent (23; 13.4%).

Model transportability was assessed separately in 150 publications. This denominator was used because those publications involved a fitted model or operational system for which geographic/site separation, temporal separation, and model state could be evaluated. Publications outside this set were not counted as negative external validations. Among the 150 transport-assessable publications, 27 were classified as strict frozen-model external, six as temporal-only, four as external with local adaptation, six as having limited site independence, 14 as conditional, and 93 as showing no demonstrated transport.

Qualitatively, the literature remains concentrated in hip, knee, and shoulder arthroplasty, with smaller signals in ankle, elbow, and thumb carpometacarpal arthroplasty. Adjacent TMJ studies were retained as contextual joint reconstruction evidence but excluded from the 172-publication arthroplasty-specific task denominator. A recent bibliometric analysis documents rapid growth in joint arthroplasty AI research and identifies implant recognition as an emerging thematic focus [37]. A broad systematic review of knee arthroplasty AI and machine-learning studies further illustrates the range of imaging, complication, implant, revision, PROM, and functional applications represented in that domain [38]; image-analysis and shoulder arthroplasty reviews similarly show breadth of application while emphasizing the need for clinical integration and comparative evidence [39,40].

The corresponding clinical engineering layers, translational signals, deployment barriers, and near-term roles are summarized in Table 3.

Figure 2 shows the distribution of the 172 core arthroplasty-specific primary-AI publications. Counts and percentages describe publication-level activity within this analytical set and should not be interpreted as independent cohorts, models, effect sizes, or clinical readiness rankings.

### 3.2. Clinical Prediction and Patient Outcomes

Clinical prediction and utilization modeling form one of the largest application families in arthroplasty AI. These models have been used to predict complications, mortality, readmission, discharge destination, length of stay, same-day discharge eligibility, revision risk, satisfaction, patient-reported outcome improvement, chronic pain, transfusion, and cost [9,24,41,42,43,44,45,46,47,48,49,50,51,52,53,54,55,56,57,58,59,60,61,62,63,64]. Their practical appeal is clear: arthroplasty is high-volume, resource-intensive, and increasingly shaped by value-based care, outpatient pathways, bundled payments, and demand for individualized counseling.

Fairness has also begun to be evaluated directly rather than assumed from aggregate performance. A large multicenter shoulder arthroplasty analysis found few predictions outside prespecified tolerance ranges overall, but residual disparities clustered in younger and underrepresented ethnic subgroups; low overall violation rates therefore did not establish universal fairness or external transportability [65].

Beyond supervised binary prediction, unsupervised learning has also been used to identify quality-of-life phenotypes after TKA, suggesting that arthroplasty recovery may be better represented by trajectory-based subgroups than by simple success–failure labels [66].

The strongest clinical prediction signals generally occur when outcomes are short-term, operationally defined, and closely linked to measurable perioperative variables. Models for same-day discharge, short length of stay, inpatient mortality, transfusion, and selected complication endpoints can provide clinically useful triage information. For example, outpatient pathway and same-day discharge models have reported discrimination in a range that may support preoperative pathway selection [9,47], while externally validated total joint arthroplasty models showed better discrimination for mortality and composite complications than for readmission [24]. Concretely, the outpatient-selection model achieved areas under the curve of 0.80 for same-day discharge after TKA and 0.81 after THA [9], whereas the externally validated total joint arthroplasty model reported an AUROC of 0.78 for 30-day mortality and 0.73 for 90-day composite complications, but only 0.61 for 30-day readmission [24]. More recent studies extend this short-term, operationally defined pattern to temporally evaluated revision complications, national-registry deep vein thrombosis (DVT) risk with calibration and decision curve analysis, early patient-controlled analgesia (PCA) discontinuation with precision–recall area, Brier score, and decision curve reporting, and Finnish-register revision/death prediction [67,68,69,70].

By contrast, models targeting satisfaction, patient-reported improvement, chronic pain, revision, and long-term benefit remain more heterogeneous. These endpoints depend not on implant choice or surgical execution alone but also on baseline symptom burden, mental health, expectations, comorbidity, rehabilitation, socioeconomic context, and health system behavior. In several studies, baseline patient-reported outcome measures and simple preoperative thresholds performed similarly to more complex machine-learning models [23,42,51,60,61,62]. For example, machine learning and preoperative score thresholds achieved nearly identical discrimination for minimal clinically important improvement after TKA, with areas under the curve of 0.77 versus 0.74 for the SF-36 physical component and 0.89 versus 0.88 for WOMAC [23]. More broadly, systematic reviews report that discrimination for these outcomes varies widely: patient-reported outcome models have spanned AUCs of approximately 0.45 to 0.97 [71], and fewer than 35.0% of predicted outcomes reached excellent or outstanding discrimination, with machine learning outperforming logistic regression in only a minority of studies [7]. That variability does not make such models unimportant, but it changes how they should be read. Their best near-term role is decision support for counseling, risk stratification, and expectation management—not autonomous decision-making.

Multimodal forecasting is beginning to reach genuinely independent evaluation. A model combining routine clinical data and radiographs retained an AUROC of 0.79 in Osteoarthritis Initiative (OAI) external validation, but weak precision–recall for surgical-timing bins and absent calibration limited its use as an individual scheduling tool [72].

The critical lesson is that arthroplasty prediction models should not be judged by discrimination metrics alone. Reported accuracy in this literature has reached 88.0% to 100.0% for some complication models [71]; for rare events, such headline figures can substantially overstate clinical usefulness. A model with acceptable area-under-the-curve performance may still have limited clinical value if it is poorly calibrated, not externally validated, difficult to interpret, or unable to change decisions. Future clinical prediction work should therefore prioritize calibration, decision curve analysis, comparison with simple baselines, external validation, and prospective workflow-impact testing.

Broader evidence reinforces these limits. A systematic review and meta-analysis of orthopedic readmission models reported only moderate average discrimination and frequent high risk of bias, with higher apparent performance often associated with single-institution data or predictors available only after surgery [73]. In shoulder arthroplasty, internal-rotation prediction achieved moderate-to-high internal performance without demonstrated external transport or calibration [74]. A single-system model for postoperative psychological distress reported moderate discrimination and calibration but remained locally derived [75]. Conventional complication scores also showed outcome-specific portability: external validation in a separate hospital was acceptable for delirium but not for several other complications [76].

### 3.3. Imaging Classification, Measurement and Implant Identification

Imaging automation and implant identification operate in bounded-task environments. The input is usually a radiograph or a computed tomography image, the output is a classification or a measurement, and the result can be checked against implant labels, geometric measurements, serial imaging, operative records, or expert review. What distinguishes these tasks is not that they perform better than others, but that a clinician receiving the output can establish whether it is correct. That property is separate from readiness for deployment, and this section reports what the studies evaluated rather than where the applications stand.

In total hip arthroplasty, deep learning and computer vision have been used to identify implant models, measure acetabular component inclination and version, quantify femoral stem subsidence, assess dislocation risk, and create radiographic registries [17,18,19,77,78,79,80,81,82]. These systems often report performance in units that are directly meaningful to surgeons, such as angular error or millimeter-level subsidence error, not just abstract classification metrics. Hip implant recognition is particularly mature, with some models reporting near-perfect discrimination in defined implant sets and newer systems incorporating uncertainty quantification and outlier detection [17,19,80,81,82]. Quantitatively, automated radiographic measurements differed from expert readings by a mean of about 1.35° for acetabular inclination [79] and 0.6 mm for femoral stem subsidence [18]. Implant-recognition models reached an AUROC of 0.999 with 99.6% accuracy across 18 hip implant designs [17], and an uncertainty-aware tool achieved 98.9% accuracy with conformal outlier detection [19]. Additional patient-level internal studies reported 91.5% stem-image accuracy across four designs and 98.9% accuracy across 12 hip/knee implant models with a five-specialist comparator [83,84]. A multimodal early-surveillance model used an independent prospective cohort, but external AUC fell to 0.67 and low specificity limited autonomous virtual follow-up [85].

In total knee arthroplasty, implant identification, loosening detection, broad implant detection, and radiograph-based classification have similarly shown strong performance [28,86,87,88,89,90]. The emergence of uncertainty-aware TKA implant classifiers is especially important because it moves the field beyond simple benchmark accuracy. A clinically credible model must know when it is correct and, just as important, when the input may be unfamiliar, low-quality, out-of-distribution, or unsafe for automated classification [28]. In reported series, knee implant detection and classification achieved AUCs up to 1.00 [86] and accuracy near 99.0% on a held-out partition from the same pooled collection [87], and loosening-detection models reached accuracies of roughly 88.0% to 96.0% [89,90]. The uncertainty-aware TKA-AID classifier reached 99.7% accuracy on its internal test set and flagged 93.0% of out-of-distribution images as outliers [28]. An intraoperative-reference loosening proof-of-concept achieved an AUC of 0.81 under five-fold internal evaluation [91]. A Conformité Européenne (CE)-marked alignment-measurement system showed excellent agreement with blinded manual measurements and explicitly suppressed outputs for unsupported implant configurations, illustrating a practical fail-safe for automated measurement [92].

Shoulder arthroplasty is following the same trajectory. Early proof-of-concept classifiers have developed into multiclass implant-recognition systems, large-scale radiographic organization tools, reverse-shoulder implant brand classifiers, and automated postoperative measurement systems [93,94,95,96,97,98,99]. Reported recognition accuracies for these systems range from about 86.0% to 97.0% across 10 to 22 implant types [93,94,97], with the best-performing models reaching AUROCs near 1.00 [97,99]. A reverse-shoulder brand classifier achieved 96.0% accuracy with conformal prediction coverage of 0.98 [95], and an automated measurement system reached interobserver agreement (ICC) of 0.86 to 0.93 for glenosphere positioning [96]. Recent shoulder models that include uncertainty quantification and rapid image processing suggest that shoulder imaging AI may become clinically relevant for revision planning, registry enrichment, and postoperative surveillance. Shoulder arthroplasty also has a growing parallel literature in tabular outcome prediction, implant size estimation, and broader shoulder/elbow AI applications, although recent reviews continue to emphasize limited external validation, reporting heterogeneity, and methodological fragility [100,101,102,103,104,105,106,107,108,109,110].

Across hip, knee, and shoulder arthroplasty, imaging AI has a clearer near-term workflow pathway than many broader prognostic applications because it addresses concrete surgical problems: identifying unknown implants before revision surgery, reducing manual radiographic measurement burden, organizing imaging archives, flagging loosening or component migration, and supporting implant-specific planning. These are not speculative uses. They are practical engineering tasks embedded in recognizable clinical workflows.

The surrounding evidence base is broader than individual classifier studies. A systematic review of implant model classification found high reported performance but substantial heterogeneity in label spaces, methods, and outcome reporting [111]. A knee-loosening classifier achieved strong internal discrimination in a surgically confirmed case-control design, but the selected spectrum and small held-out set limit inference about early diagnosis or portability [112]. Adjacent multicenter evidence from automated Kellgren–Lawrence grading illustrates how a model trained on United States cohorts can be assessed in Korean tertiary hospitals with both discrimination and Brier score reporting [113]. A prospective post-TKA wound-imaging protocol provides a useful example of planned standardized image acquisition and blinded clinical labeling, although empirical results are not yet available [114]. An exploratory implant-identification study also proposed complementary CNN and multimodal LLM roles, but the LLM component was not evaluated as a diagnostic classifier [115].

### 3.4. Templating, Sizing, Planning and Execution Support

Planning-oriented AI occupies a critical position between prediction and intervention. Unlike broad risk models, templating and sizing tools directly influence preoperative preparation, implant inventory, surgical planning, and robotic workflows. That places them squarely within clinical engineering, since they connect computational inference to physical surgical execution.

In total knee arthroplasty, implant sizing and templating form one of the strongest planning subfields. Demographic-based and radiograph-based models have repeatedly achieved high within-one-size accuracy, even when exact-size prediction remains more difficult [22,116,117,118,119,120]. In a systematic review, within-one-size accuracy ranged from 88.3% to 99.7% for femoral and 90.0% to 99.9% for tibial components [120], whereas exact-size accuracy was more variable, from roughly 40.0% in earlier demographic models [22] to about 89.0% in recent deep-learning radiographic models [117]. The distinction matters clinically. Exact implant size prediction is desirable, but within-one-size performance may still reduce planning variability, improve inventory preparation, and decrease dependence on manual templating. Planning-oriented TKA AI also includes models for templating mismatch and sagittal component-position optimization, illustrating that knee planning tools increasingly address component size together with the reliability and functional implications of the preoperative plan [2,121].

In total hip arthroplasty, AI-assisted templating and three-dimensional planning are increasingly important. Studies have explored planning reliability, press-fit stability, radiograph-based templating, and AI-driven three-dimensional planning for implant positioning and reconstruction fidelity [122,123,124,125,126]. Recent meta-analytic evidence suggests that AI-assisted three-dimensional preoperative planning may outperform conventional two-dimensional templating for exact acetabular cup and femoral stem size prediction [126]. In that meta-analysis, AI was significantly more accurate than two-dimensional templating for exact acetabular cup size (odds ratio 3.85, 95% confidence interval 2.79 to 5.32) [126]; separately, a radiomics model predicted intraoperative cup press-fit stability with an AUC of 0.823 [123], and a planning accuracy model reached 97.9% classification accuracy for the acetabular cup [122]. That shifts the discussion from convenience to reconstruction quality.

Clinical evaluations of AI-HIP and related three-dimensional systems show a consistent but bounded pattern: shorter planning time and higher exact or within-one-size component agreement than two-dimensional templating, while most studies remain single-center, platform-specific, and unable to demonstrate durable functional benefit [127,128,129,130]. A small randomized-envelope THA trial reported better component size prediction and shorter surgery [131]; the platform-development study reduced automated three-dimensional processing time but did not establish independent external validation or superior clinical outcomes [128].

Robotics-linked planning and segmentation represent the next logical step. In robotic-assisted TKA, machine-learning models have incorporated surgical planning variables to predict functional improvement, while deep-learning segmentation models have improved knee CT segmentation for robotic workflows [132,133]. Additional work on alignment determination from full-length radiographs and three-dimensional imaging-based sizing further suggests that planning AI is moving toward image-rich, geometry-aware, and platform-integrated surgical support [134,135]. In these efforts, a robotic TKA model predicted functional improvement with an AUC of 0.80 [132], and an automated alignment tool measured postoperative limb alignment within a mean error of 0.08° for the hip–knee–ankle angle and under 1° for individual component angles, in less than 0.1 s per image [134].

Clinical planning and execution studies show why algorithmic contribution must be separated from the surrounding intervention. In younger TKA patients, AI planning shortened preparation time without improving ±1-size component prediction [136]. AIJOINT combined rapid segmentation and patient-specific instrumentation (PSI) design with a small retrospective clinical cohort [137], whereas AI-planned robotic TKA improved sizing and selected early perioperative measures but could not isolate AI from the combined robotic workflow [138]. AI-designed PSI improved resection and component-position accuracy in THA without a short-term patient-reported outcome advantage [139]; in a randomized TKA trial, both arms used AI planning, so the between-group effect isolated PSI-guided execution rather than AI planning itself [140].

The translational implication is that planning AI should be evaluated differently from generic prediction AI. The key question is whether the model improves operative preparation, component selection, alignment strategy, implant inventory, operative efficiency, reconstruction accuracy, or patient outcome—not merely whether it predicts correctly. Future studies should therefore report clinically actionable planning endpoints, not just algorithmic performance.

Supporting syntheses and newer clinical series point in the same direction. A systematic review and meta-analysis found better exact component size agreement and fewer major sizing deviations with AI-assisted or three-dimensional THA planning than with conventional two-dimensional templating, while clinical score differences were small and software/geographic concentration limited generalizability [141]. A structured narrative review similarly reported frequent cup-angle errors below 3°, component size agreement around 80.0–85.0%, and shorter planning time, but emphasized image artifacts, limited generalizability, and weak linkage to intraoperative execution [142]. Conventional CT-based computer-aided design provides a useful non-AI comparator, with improved sizing agreement over radiograph-based planning in a small cohort but insufficient allocation detail for causal conclusions [143]. Additional single-center studies reported high exact component size agreement for AI-based TKA planning [144] and high component agreement in a small revision-THA AI-HIP series [145]; these findings remain platform- and setting-specific.

### 3.5. Data Extraction, Registry and Documentation Infrastructure

Text extraction addresses a problem the rest of arthroplasty artificial intelligence depends on, and it is strategically important for that reason rather than because it is the most mature domain. Many of the most valuable arthroplasty variables are simply not available in structured databases. Surgical approach, fixation, bearing surface, laterality, constraint, implant model, revision indication, periprosthetic fracture classification, and nuanced postoperative findings are often embedded in operative notes, radiology reports, scanned documents, and other free-text records. Without reliable extraction, multimodal models and registry-based research inherit whatever the coded fields happen to contain.

Arthroplasty-specific NLP studies report high accuracy in extracting common data elements from total hip and total knee arthroplasty operative notes, identifying and classifying periprosthetic femur fractures, and retrospectively collecting registry or quality-review variables from unstructured charts [20,21,146,147]. Extraction of common data elements from operative notes achieved 99.2% accuracy for surgical approach in hip notes [20], and an F1 score of 99.9% for implant model in knee notes [21]; periprosthetic fracture detection reached 100.0% sensitivity with 99.8% specificity [146], and registry variable extraction reached 96.3% overall accuracy [147]. These figures describe performance at or near the developing institution. Where a study reports performance both on arrival at a new site and after local adaptation, the two differ: out-of-box accuracy at a second institution was 96.6% for surgical approach and 95.7% for fixation, rising to 99.0% and 98.0% after site-specific refinement [148], and an independent center reached 100.0% for all three elements only after two small sources of systematic error, both involving local dictation styles, were identified and corrected [149]. The distinction matters for interpretation rather than for judgment: in the latter case the refinement took under an hour, and the difference in document pipeline generated no error at all, so the adaptation cost was low. What the headline figures describe is performance after local adaptation, not performance on arrival, and reporting the two separately is what allows a receiving site to know which it should expect.

Newer work also compares locally hosted language models with rules-based extraction. A LLaMA pipeline improved bearing-surface extraction from 74.0% to 89.0%, but was roughly two orders of magnitude slower and still produced clinically plausible hallucinations, showing that higher extraction accuracy does not remove operational and safety trade-offs [150].

The core insight is that NLP works less as a prediction tool than as a data-conversion technology, turning narrative clinical documentation into structured, registry-ready variables. Without this infrastructure, many future multimodal AI systems in arthroplasty will be constrained by incomplete or poorly structured datasets.

Text extraction sits underneath much of the rest of this field rather than alongside it. It can support registry construction, multicenter research, quality improvement, implant surveillance, infection phenotyping, revision risk modeling, and the integration of imaging, laboratory, operative, and patient-reported data. Where extraction is unreliable, whatever is built on it inherits that unreliability, which is a dependency rather than a level of maturity.

A contemporaneous commentary on LLM-assisted registry curation reaches a similar conclusion: the approach is promising, but infrastructure, privacy, standardization, and explicit governance remain prerequisites for routine use [151].

### 3.6. Periprosthetic Joint Infection and the Reference-Standard Problem

PJI is one of the most clinically important proving grounds for arthroplasty AI. It combines high morbidity, diagnostic uncertainty, costly consequences, revision complexity, evolving definitions, culture-negative cases, laboratory biomarkers, operative findings, and longitudinal outcomes. For this reason, infection-focused AI is best seen as a test case for whether multimodal decision support can improve high-stakes arthroplasty care.

Current PJI-related AI studies span diagnosis, postoperative infection prediction, recurrent infection after revision, biomarker-based probability scoring, diagnostic certainty, cost modeling, and mortality stratification after revision for infection [25,26,27,33,50,152,153,154,155,156,157,158]. Some models compare machine-learning approaches with established consensus-based scoring systems, while others use biomarker-rich or electronic health record-based inputs to generate individualized probability estimates. Reported discrimination varied widely across these distinct tasks: diagnostic models reached AUCs as high as 0.99 [25] but as low as 0.73 in other cohorts [152], infection prediction after primary total hip arthroplasty reached an AUC of 0.88 [50], recurrence and post-revision infection models showed heterogeneous moderate-to-good discrimination in the available reports [26,154], and mortality stratification after revision for infection reached an AUROC of about 0.86 [158]. A systematic review of diagnostic models reported AUCs spanning 0.68 to 0.99, underscoring that performance does not transfer uniformly across PJI use cases [155].

A synovial-fluid probability model achieved high same-laboratory temporal performance and reduced inconclusive classifications, but biomarker-derived labels and overlap with the modified ICM comparator limited inference against an independent diagnostic truth [159]. A four-modality prosthetic-loosening model improved three-class performance over single modalities, yet PJI recall remained 0.60 in a 15-case validation class, and no external validation or calibration was reported [160].

The domain has several features that make it attractive for AI development. The clinical problem is consequential, the decision pathway is complex, and the data are naturally multimodal. A useful PJI-support system could theoretically combine serum markers, synovial biomarkers, microbiology, operative history, imaging, prior revision burden, comorbidity, antibiotic exposure, pathology, and free-text clinical documentation.

However, PJI also illustrates why high-performance metrics must be interpreted cautiously. Diagnostic labels depend on consensus definitions, culture status, biomarker availability, and institutional practice. Recurrence prediction, primary infection prediction, diagnostic classification, and mortality stratification are related but distinct tasks. Therefore, performance estimates should not be generalized across PJI use cases without careful attention to the target definition, dataset, and clinical context.

The most credible future role of PJI AI is to reduce diagnostic ambiguity, identify high-risk phenotypes, support revision planning, and standardize multimodal interpretation—not to replace infectious-disease or arthroplasty expertise. Prospective validation using standardized definitions is essential before such systems can be trusted in clinical pathways.

The same transport problem is visible in established non-AI risk scores. Multinational external validation of preoperative PJI models retained only moderate discrimination and showed clinically relevant miscalibration at higher predicted risks, illustrating why geographic evaluation and calibration should be considered together [161]. A fracture-related hip arthroplasty PJI model also reported stable same-center temporal performance with explicit calibration, providing temporal rather than geographic evidence [162].

### 3.7. Patient and Clinician Communication, Education and Consent

LLMs and chatbots are among the fastest-growing and most visible areas of arthroplasty AI. Studies have evaluated ChatGPT and related systems for patient education, frequently asked questions, informed consent, postoperative counseling, multilingual engagement, online information support, and comparison with surgeon- or nurse-generated responses [13,14,15,16,163,164,165,166,167,168,169,170,171,172,173,174]. Newer studies broaden that scope to custom perioperative care aids, cross-language reliability, retrieval-augmented supervised consent, and direct model-to-model comparisons [175,176,177,178].

The overall pattern is consistent. LLMs can often generate readable, fluent, and broadly acceptable answers to common arthroplasty questions. Some studies suggest that patients may perceive AI-generated responses as useful or comparable to clinician responses in selected contexts, and early interventional work suggests possible benefits for anxiety reduction during consent support [14,16,166,167,168,171]. In these studies, ChatGPT responses were rated appropriate at rates similar to nurse responses (73.3% for each in one comparison [167], and 95.0% versus 93.3% in another [168]), achieved a mean physician-rated accuracy of 4.26 out of 5 [14], and, in a randomized consent study, were associated with lower preoperative anxiety (HADS-A 10.48 versus 12.75, P = 0.04) [16]. Multilingual chatbot studies also raise an important equity-related possibility: supervised AI communication tools may help reach patients with limited English proficiency or limited access to perioperative education resources [15]. That opportunity is not uniform across languages: in a 10-question THA pilot, expert clinical-reliability ratings were lower for Spanish than English across ChatGPT-4o, Gemini 2.0 Flash, and Microsoft Copilot [176]. In doctor-supervised THA consent, retrieval augmentation reduced hallucinations from 38.0% to 10.0%, while patients continued to prefer physician-led consent [177].

At the same time, the limitations are equally important. Fluency is not reliability. LLM outputs may be incomplete, overconfident, variable, insufficiently individualized, poorly referenced, or unsafe when used outside a supervised setting. Consistent with this, only 59.2% of ChatGPT responses in one study were judged acceptable as a sole information source [14], and in another only 2 of 10 responses required no correction [13]. Even when responses are rated as acceptable, they do not replace shared decision-making, individualized risk discussion, or surgeon accountability.

Therefore, the correct interpretation is not that LLMs are ready to function as autonomous arthroplasty information systems. Their more defensible role is as supervised communication infrastructure: drafting patient-facing explanations, answering common low-risk questions, supporting multilingual engagement, reinforcing discharge instructions, and preparing patients for clinician-led discussion. Safer deployment will require specialty-specific content boundaries, retrieval-augmented knowledge bases, clinician review, hallucination safeguards, escalation pathways, and clear medicolegal accountability.

Recent studies illustrate both the opportunity and the limits of this supervised role. Retrieval-augmented systems improved performance on orthopedic guideline questions and in a deployed patient education chatbot, although the latter remained sensitive to retrieval quality and was not arthroplasty-specific [179,180]. LLMs also showed potential for the expert-supervised preliminary stratification of TKA educational videos [181]. In postoperative TKA instructions, four general-purpose models produced highly accurate answers in a small standardized benchmark, but readability remained above ideal patient-facing levels, and only 12 outputs were compared [182]. A separate blinded TKA benchmark found high but closely clustered model performance, fair rater agreement, and version-related instability, supporting cautious interpretation of rankings [183].

### 3.8. Utilization and Workflow Optimization

Workflow optimization is a smaller but strategically important part of arthroplasty AI because it shifts evaluation from prediction metrics to system performance. In clinical AI studies, model success is often framed around discrimination, accuracy, or F1 score. In workflow AI, the more relevant endpoints include operating room utilization, overtime, underutilization, case throughput, staff burden, implant inventory, scheduling accuracy, patient flow, and resource allocation.

Disposition and utilization models now include direct comparison with the American College of Surgeons (ACS) Risk Calculator, independent institutional validation, and fairness-aware NLP augmentation [184,185,186,187]. The strongest transport examples retained discrimination, calibration, and net benefit in an independent institutional cohort [185,186]; by contrast, the fairness-aware model used an internal case-level split and combined emergency department (ED) visits, readmission, and skilled-nursing-facility discharge in one endpoint [187].

Several arthroplasty-adjacent studies already point in this direction. Operative-time prediction models in TKA and shoulder arthroplasty address the practical problem of scheduling accuracy and operating room efficiency [59,106]. For instance, an operative-time model in primary TKA reached an AUC of 0.82 for prolonged operative time [59], and a shoulder arthroplasty duration model predicted case length accurately in 79.7% of cases, compared with 64.1% for human schedulers [106]. Robotics-linked segmentation and planning models connect computational outputs to procedural execution [132,133]. A predict-then-optimize elective orthopedic scheduling model trained on large THA and TKA datasets demonstrated that machine-learning-based scheduling can reduce overtime, although this may come with trade-offs such as increased underutilization [188]; in the study, the underlying duration prediction models were accurate within a 30-min buffer in 78.1% of TKA and 75.4% of THA cases [188]. A later temporal-test predict-then-optimize study similarly projected fewer idle operating room hours and fewer operating room days, with a small simulated overtime trade-off; these remain modeled operational effects rather than observed deployment outcomes [189].

The translational message here is blunt: implementation studies should not present a single favorable metric as proof of value. A scheduling model that reduces overtime but increases underutilized time may still be useful, but only if the trade-off is explicit and aligned with institutional priorities. Similarly, an implant-recognition tool may be highly accurate, but the clinically meaningful question is whether it reduces revision-planning time, prevents wrong implant inventory, or improves intraoperative preparedness.

Workflow AI should therefore be evaluated using operational endpoints that matter to surgeons, hospitals, and patients. These include time saved, errors prevented, resources optimized, cases completed, delays reduced, and clinical teams supported.

Economic evidence should be separated by design. A 17-week United Kingdom National Health Service (NHS)-perspective decision model estimated £450.56 lower cost and 0.02 additional quality-adjusted life-years (QALYs) for an AI-assisted rehabilitation pathway, but the result depended partly on a small trial, expert opinion, and model assumptions and therefore represents modeled cost-effectiveness rather than observed real-world savings [190]. That move takes arthroplasty AI from algorithm benchmarking toward engineered system performance.

Adoption evidence also shows that technical capability does not determine implementation by itself. In a survey of Flemish surgeons already using robotic TKA, most reported changes in alignment or constraint strategy, while cost remained a major concern and fewer than half expected improved clinical outcomes [191].

### 3.9. Evidence Distribution by Anatomical Scope

The supporting literature is heavily concentrated in hip, knee, and shoulder arthroplasty. That concentration likely reflects procedure volume, imaging availability, registry infrastructure, commercial interest, and the maturity of existing research ecosystems. However, it also means that the phrase “AI in arthroplasty” should not be interpreted as equally applicable across all joints.

Total ankle arthroplasty has the strongest signal among non-hip, non-knee, and non-shoulder domains. The ankle literature includes AI-assisted or digital-twin concepts for personalized tibiotalar alignment, length-of-stay prediction, long-term outcome prediction using CT-derived features, patient-facing ChatGPT evaluation, social media sentiment analysis, and implant design optimization [164,173,192,193,194,195,196]. Where performance was reported, a length-of-stay model reached an AUC of about 0.73 [192] and a CT-feature model predicted 24-month outcomes with an AUC of 0.90 in validation [193]. Taken together, the breadth suggests ankle arthroplasty may be the first smaller-volume domain to develop a meaningful AI ecosystem.

Total elbow arthroplasty and thumb carpometacarpal arthroplasty remain much less mature. Current signals include frailty-based adverse-outcome prediction, patient information studies, short-term complication prediction, and postoperative function prediction [165,197,198,199]. Reported discrimination was modest and task-dependent: a frailty-based model reached AUROCs of 0.85 for length of stay and 0.78 for non-home discharge after elbow arthroplasty [197], whereas thumb-arthroplasty models predicted hand function (AUC 0.74) better than pain (AUC 0.59) [199] and reached only 0.55 to 0.61 for short-term complications [198]. These studies are important because they show diffusion of AI into smaller reconstructive domains, but they do not yet support broad claims of clinical readiness.

The temporomandibular joint is the only non-orthopedic joint covered in this review. It is included because that joint is itself treated by alloplastic total joint replacement, and because the AI methods applied to it (segmentation, osteoarthritis grading, and landmark or feature recognition on CT and MRI) closely parallel those used in limb arthroplasty; it is therefore presented as an adjacent illustration of how the same imaging-engineering techniques are diffusing into reconstructive joint surgery beyond the orthopedic skeleton. Adjacent temporomandibular joint AI literature was retained only as contextual joint reconstruction evidence. TMJ studies show active development in diagnostic imaging, osteoarthritis classification, arthropathy detection, disc displacement analysis, and radiographic feature recognition [200,201,202,203,204]. However, these studies should not be treated as core arthroplasty evidence and should not be used to infer readiness of AI tools in hip, knee, shoulder, ankle, elbow, or hand arthroplasty.

### 3.10. Cross-Domain Patterns in Translation and Deployment Evidence

Across tasks, technical performance, evaluation outside the development setting, workflow evaluation, and demonstrated clinical benefit represent different evidence properties. A publication may satisfy one without satisfying the others; they are therefore reported separately rather than combined into a single maturity score.

Bounded tasks permit clearer verification. Implant identification, radiographic measurement, component sizing, operative-note abstraction, and scheduling models produce outputs that can be checked directly against images, measurements, source documents, or operational records. Broader prognostic tasks and patient-facing language models are harder to verify at the point of use and depend more heavily on calibration, governance, and prospective evaluation.

Figure 3 summarizes six criterion-specific signals across the eight task groups. The denominators are intentionally criterion-specific. Geographic/site and temporal columns use the 139 core publications for which transportability was assessable, whereas the other columns use task-specific eligibility rules. Planning/sizing/execution and NLP/data infrastructure contained the largest relative signals for geographic/site-separated evaluation (12/27 and 4/12, respectively). Later non-overlapping temporal evaluation remained sparse in every task group, indicating that time-separated testing was uncommon even where site-separated evidence was available.

Performance-completeness and decision-utility assessment was restricted to prediction-oriented tasks for which calibration or overall-error measures were applicable; it was not expected from segmentation, measurement, retrieval, or many communication studies. Positive performance-completeness signals were most visible in clinical prediction and PJI studies. Uncertainty/out-of-distribution (OOD) handling was uncommon and concentrated in selected implant-identification and data-infrastructure applications. Observed workflow or patient benefit evidence was also limited and appeared mainly in planning/execution and selected communication or operational studies.

The bands in Figure 3 are descriptive visual aids. The underlying *n/N* values are the primary results, and columns based on confirmed-present lower bounds should not be interpreted as definitive absence rates among the remaining publications. Small task groups are retained because limited evidence is itself informative; when the eligible denominator was below four, *n/N* is shown without a categorical band.

Taken together, the pattern supports clinician-supervised, auditable applications rather than a single autonomous model intended to replace surgical judgment.

## 4. Discussion

### 4.1. Principal Interpretation

The synthesis here reframes artificial intelligence in arthroplasty as a system engineering problem, not a contest between algorithms and surgeons. The key finding is that the translational value of these applications differs substantially by task, even as their number grows. Across the supporting literature, the most credible near-term applications are bounded, verifiable, and workflow-adjacent: implant identification, automated radiographic measurement, component sizing, operative-note abstraction, registry-ready data extraction, selected PJI-support models, and workflow-optimization tools. By contrast, broad prognostic models for satisfaction, PROM improvement, chronic pain, readmission, revision, and long-term outcome prediction remain more heterogeneous and less consistently ready for implementation [1,3,6,7,10,23,71].

This distinction changes the central question. It is not whether AI can replace surgical judgment. Arthroplasty involves preference-sensitive decisions, technical execution, implant-specific reasoning, intraoperative adaptation, patient expectations, rehabilitation behavior, and long-term biological uncertainty. These features cannot be reduced to a single model output. The more realistic and clinically meaningful question is whether AI can be engineered into the arthroplasty pathway as accountable precision-support infrastructure. In that role, AI may strengthen surgical care by making imaging, planning, documentation, infection assessment, communication, and workflow more measurable, scalable, and reproducible, while keeping responsibility clinician-led.

### 4.2. Why Bounded Tasks Are Easier to Evaluate than to Deploy

The clearest translational gradient in the literature is the difference between bounded technical tasks and broad patient-centered prediction. Implant identification, cup-angle measurement, femoral stem subsidence quantification, TKA component sizing, shoulder implant classification, and operative-note abstraction have relatively clear inputs, outputs, and verification standards [17,18,19,20,21,22,28,77,78,79,80,81,82,87,88,89,90,95,96,98,116,117,118,119,146,147,148,149,205]. A radiographic implant classifier can be checked against operative records or known implant labels. A cup-angle tool can be compared with expert measurements. An NLP abstraction pipeline can be validated against manually reviewed operative notes. These tasks are therefore easier to benchmark, easier to audit, and easier to place into human-in-the-loop workflows. Reported performance in these bounded tasks is correspondingly high and directly auditable, with implant-recognition AUROCs near 0.99, a mean acetabular inclination error of 1.35°, and a femoral stem subsidence error of 0.6 mm [17,18,79].

In contrast, patient satisfaction, PROM improvement, chronic pain, revision risk, and readmission are not purely technical targets. They are shaped by baseline symptoms, expectations, psychological status, comorbidity, rehabilitation, social determinants, access to care, institutional practice patterns, and follow-up behavior. Models in these areas therefore often show variable performance and sometimes fail to outperform simpler baselines such as preoperative PROM thresholds or conventional regression models [23,42,51,60,61,62,63]. The lesson is not that broad prognostic modeling is unimportant. Rather, such models should be treated as counseling and triage aids, not as autonomous decision engines.

The implication for study design is direct: arthroplasty AI papers should not treat all AUC values as equivalent. An AUC of 0.80 for a narrow radiographic classification task is not conceptually equivalent to an AUC of 0.80 for a complex behavioral or long-term clinical outcome. The former may support a concrete workflow action; the latter may require extensive calibration, interpretability, decision curve analysis, and prospective testing before it can safely influence care.

### 4.3. Discrimination, Calibration and Decision Utility

Clinical prediction remains one of the largest and most visible areas of arthroplasty AI. Models for mortality, complications, transfusion, length of stay, discharge destination, same-day discharge, readmission, satisfaction, PROM improvement, and revision risk can support preoperative counseling, pathway selection, resource planning, and risk communication. However, the literature suggests that prediction alone is not enough. A model that predicts an outcome does not necessarily improve care unless its output changes a decision, reduces uncertainty, saves time, prevents an error, or improves patient understanding [9,24,41,42,43,44,45,46,47,48,49,50,51,52,53,54,55,56,57,58,59,60,61,62,63,64,206].

Calibration is a hierarchy, not a single property. Four nested levels have been defined: mean calibration, in which average predicted risk matches average observed risk; weak calibration, in which intercept and slope are correct; moderate calibration, in which the event rate among patients assigned a given predicted risk equals that risk; and strong calibration, which requires this for every covariate pattern [207]. Moderate calibration is the level at which non-harmful decision-making is guaranteed.

Several studies report evidence at the first level and describe it in terms appropriate to a higher one. One reports a calibration intercept without a slope and characterizes calibration as excellent [49]. Another reports standardized incidence ratios close to unity and applies the same description [57]. Both statements concern mean calibration.

Where slopes are reported, they often reveal miscalibration that discrimination does not. A slope below one indicates overfitting and predictions that are too extreme; a slope above one indicates underfitting and predictions that are insufficiently extreme [207]. Values of 2.41 [206], 2.431 [208], and 14.9 [50] were accompanied by descriptions of calibration as adequate or excellent. A later transfusion model reported slopes of 7.10–8.15 with intercepts around −3 despite favorable calibration language [209]. One study also reported a negative slope [208]; this indicates an inverse relation between predicted risk ordering and observed risk and cannot be corrected by ordinary positive-slope recalibration without reversing the ordering.

Rare-event mortality prediction provides the converse warning: a very small absolute Brier score can coexist with substantial slope miscalibration. In revision arthroplasty, a model with Brier score 0.005 reported a calibration slope of 0.54, consistent with predictions that were too extreme despite favorable discrimination [210].

Studies reporting the complete apparatus are present and instructive. One reports slopes with confidence intervals for five models spanning 0.85 to 1.25 alongside a null-model Brier score [53]; another reports slopes, intercepts, and Brier scores with intervals for three models and a null comparator [26].

Null comparators, where reported, were exceeded only narrowly. Five studies made a comparison against a model using no patient information [26,53,59,211,212]. In one low-prevalence PJI study, the best model achieved an area under the curve above 0.95 while its Brier score exceeded a constant-risk benchmark derived from the reported event prevalence [208]. The benchmark is a project-derived comparator, not a null model reported by that paper. These examples show that discrimination and probability quality can diverge substantially.

Prevalence-aware reporting also exists and is stated as such: area under the precision–recall curve was emphasized for relatively rare outcomes [54], a scaled Brier score was referenced to the null [212], and balanced accuracy was emphasized because of class imbalance [208]. Where such statements are absent, headline accuracy requires comparison with the majority-class rate before interpretation.

These observations reinforce a higher translational standard. Among 172 core primary-AI records, calibration was confirmed in 27 (15.7%), Brier score in 10 (5.8%), and decision curve or net-benefit analysis in eight (4.7%). These are confirmed-present lower bounds rather than absence prevalence. Prediction studies should report design-specific evaluation, comparison with simple baselines, calibration, and decision utility; prospective work should test whether the output changes counseling, pathway selection, workload, error rates, utilization, or shared decision-making.

Implementation-readiness hierarchy. In interpreting arthroplasty AI studies, technical performance should be separated from clinical utility. The implementation pathway used in this review progresses from technical feasibility and internal validation to independent geographic, temporal, or site-separated evaluation; comparator, calibration, and decision-utility reporting; explainability, uncertainty, and failure-mode handling; workflow integration; prospective clinical or operational impact; and governance, monitoring, and local audit. This staged interpretation is summarized in Table 4. Most current arthroplasty AI studies remain in the earlier technical feasibility, internal validation, or limited external-validation stages, whereas routine deployment requires evidence of workflow integration, impact, and ongoing governance.

Model performance and system performance are different endpoints, and only the second answers the implementation question. Discrimination, accuracy, sensitivity, specificity, and F1 describe how well a model separates cases. They do not say whether anything changed for the surgeon, the patient, or the service. A scheduling model that reduces overtime while increasing underutilized theater time may still be worth deploying, but only if both effects are reported; a single favorable metric offered as proof of value conceals the trade-off rather than resolving it. The operational endpoints that would settle the question—time saved, errors prevented, delays reduced, cases completed, resources released—are reported far less often than the discrimination statistics that cannot answer it.

### 4.4. Evaluation Outside the Development Setting

Imaging, implant identification, planning, NLP, and prediction systems may all have clinically plausible uses, but technical usefulness and evaluation outside the development environment are separate questions. A model can support implant recognition, radiographic measurement, component sizing, or information extraction while remaining untested in a distinct clinical setting.

The operational framework used here therefore records three properties separately: geographic/site independence, later non-overlapping temporal evaluation, and model state at evaluation. This avoids forcing different designs into a single binary external-validation field. It also permits studies to be described according to their design without implying that the terminology used by the original investigators was inappropriate within their own context.

Several recurring patterns were identified. Random partitions of pooled multi-institutional collections provide internal holdout evidence rather than site-independent evaluation. Multicenter development data demonstrate diversity of the development sample but do not by themselves show transport to a held-out institution. Later same-site cohorts provide temporal evidence. Evaluation on new data after renewed feature selection or model fitting represents redevelopment rather than transport of the original fitted model. Site-held-out evaluation within one health organization provides stronger separation than a random split but remains distinct from evaluation in an independent organization. By contrast, application of a fitted model without local retraining at institutions that contributed no development data meets the strict frozen-model geographic definition.

During source review, the terminology used in eight publications mapped to a different operational category under this framework. This finding is presented as a classification issue rather than a criticism of computational conduct. In most cases, the Methods section described the design sufficiently for transparent reclassification, and several publications explicitly acknowledged their own limitations. A publication-level mapping flag and concise explanation are provided in Supplementary Table S1B, and Table 5 summarizes the principal design patterns with representative examples.

Related examples show why the framework is useful beyond the illustrative table. Registry-based external validation of published conventional THA prediction models showed that variable availability limited which models could be evaluated and that calibration remained problematic even when discrimination was acceptable [216]. Same-center later-year validation of a PJI model also demonstrates that temporal evidence can be informative when it is reported separately from geographic transport [162]. Commercial planning systems were evaluated across several clinical centers [217,218,219,220,221,222,223,224,225]; these publications were retained as deployment evidence with lineage qualifiers because model/version continuity and independence were not uniform across the series.

Publication-level strict external evaluation was identified in 27 publications. Both same-site/window pairs were contained within the 12-publication commercial planning program cluster. Accordingly, the pair-level and program-level sensitivity analyses were alternative rather than cumulative: the former reduced the count from 27 to 25, whereas the latter treated all 12 program publications as one node and reduced the count directly from 27 to 16 (27 − 12 + 1). These alternative analyses show why publication-level and program-sensitive results should be presented together.

Commercial planning systems were evaluated across several clinical centers, including a series of AI-HIP studies and a Mako robotic workflow [217,218,219,220,221,222,223,224,225]. Repeated publications from one product program should not be interpreted as independent replication of 12 different models. Exact model or software-version continuity was often not reported, and some publications shared sites, time windows, authors, or vendor relationships. These records remain valuable deployment evidence, but the level at which they are counted must be explicit. A registry-based assessment of published THA prediction models provides a complementary perspective: only a small subset could be externally evaluated because required variables were unavailable, and the evaluable models showed important calibration limitations [216].

### 4.5. Local Accuracy and Demonstrated Portability

Natural language processing may be one of the most undervalued domains in arthroplasty AI. While computer vision and prediction models receive more attention, NLP addresses a deeper infrastructure problem: many of the most important arthroplasty variables are still embedded in free text. Operative approach, fixation method, bearing surface, implant model, laterality, revision indication, constraint type, periprosthetic fracture classification, and nuanced postoperative findings are often not reliably available in structured datasets [20,21,146,147,148,149,205,226].

Arthroplasty-specific NLP studies show that operative notes, radiology reports, scanned documents, and registry-related text can be transformed into structured, research-ready variables with high accuracy in selected tasks [20,21,146,147,148,149,205,226]. For example, common-data-element extraction at development sites reached accuracies of 99.0% or higher [20,21]. Cross-center use was also demonstrated, but the strongest 99.0–100.0% figures followed local refinement rather than out-of-box transport: one external site improved from 96.6%/95.7% to 99.0%/98.0% after site-specific refinement [148], and another reached 100.0% only after local dictation-style errors were identified and corrected [149]. NLP, in other words, is less a prediction tool than a data-engineering technology. Without structured, scalable, and validated data extraction, future multimodal arthroplasty AI will remain limited by incomplete registries and fragmented documentation.

The next stage is multicenter NLP infrastructure: harmonized arthroplasty data dictionaries, institution-specific validation, error auditing, integration with registries, and linkage with imaging, laboratory, implant, and PROM data. LLM-assisted extraction may expand this field further, but it should be validated against human-reviewed gold standards before being used for registry construction or clinical decision support [148,149,205,226,227,228].

### 4.6. Language Models: What Was Evaluated and What Was Not

Large language models and chatbots are becoming highly visible in arthroplasty because they are easy to access, easy for patients to understand, and capable of generating fluent responses to common questions. Studies in hip, knee, shoulder, elbow, and ankle arthroplasty suggest that LLMs can provide broadly acceptable responses for frequently asked questions, patient education, postoperative counseling, informed consent support, and multilingual engagement [13,14,15,16,163,164,165,166,167,168,169,170,171,172,173,174]. In comparative studies, surgeons rated chatbot and clinician responses appropriate at similar rates, roughly 73.0% to 95.0%, yet only about 59.0% of chatbot responses were judged acceptable as a sole information source [14,167,168].

However, fluency should not be mistaken for safety. LLM responses may be incomplete, overconfident, poorly individualized, variably referenced, or inappropriate for specific clinical scenarios. Even when patients perceive these outputs as helpful, the systems do not replace shared decision-making or individualized surgical counseling. Their safest near-term role is as supervised communication infrastructure: drafting explanations, reinforcing postoperative instructions, supporting multilingual access, preparing patients for clinician-led discussions, and answering low-risk repetitive questions within defined boundaries.

Direct verification of real-world recommendations exposes a different failure mode. In one four-city TKA referral study, only 49 of 74 recommendations were appropriate, six named surgeons were hallucinated, 13 real surgeons did not perform TKA, and only 17 of 74 contact details were accurate; appropriateness ranged from 24 of 24 recommendations in New York to seven of 16 in Lynchburg [229]. These results separate dimensions that a single accuracy label would collapse: whether the clinician exists, performs the relevant procedure, is geographically appropriate, and can actually be contacted.

For LLMs to become clinically useful in arthroplasty, they require specialty-specific guardrails: retrieval-augmented knowledge bases, institutionally approved content, hallucination safeguards, escalation pathways, readability targets, clinician oversight, documentation of AI involvement, and clear medicolegal responsibility. Without such controls, patient-facing LLMs risk amplifying misinformation under the appearance of clarity.

### 4.7. Engineering Controls and Failure-Mode Handling

The evidence reviewed here supports a practical deployment principle: arthroplasty AI should be designed around human-in-the-loop control. That does not mean simply placing a surgeon at the end of an automated pipeline. It means engineering the system so that model outputs are interpretable, uncertainty is visible, failure modes are anticipated, and escalation pathways are defined.

Safe deployment requires several engineering controls. Imaging tools should include image-quality checks, out-of-domain detection, uncertainty quantification, and implant class coverage reporting. Prediction models should include calibration, external validation, and comparison with clinically simple baselines. NLP systems should undergo local documentation validation and periodic error auditing. LLM tools should use constrained knowledge bases, clinician oversight, and escalation triggers. Workflow models should report trade-offs, not just improvements. These controls convert AI from a black-box model into an accountable clinical component.

The predominant pattern among implant-identification studies was closed-set classification. Fixed label spaces ranged from three hip stems [230], through six manufacturer knee sets [231,232], to 38 primary and revision designs [233]. Serial radiographs with image-level splitting and mixed internal/web-image subsets further blur the distinction between memorized visual similarity and independent generalization [232,233]. A closed-set classifier presented with an implant outside its label space does not report uncertainty; it assigns the nearest known class. In a revision-planning context, where the purpose of identification is to know what will be encountered, that behavior is the failure mode that matters, and it is the one the reported accuracy cannot describe. An external shoulder study also evaluated a platform across a prosthesis not represented in its development setting, illustrating why product/domain independence is a separate transport question [234].

Several records are built to detect that condition rather than to force a label. Three pair conformal prediction with explicit outlier or domain detection [19,28,235], while a fourth uses conformal prediction without a separately described outlier detector [95]. The two most fully characterized implant classification systems retained high closed-set performance—98.9% internal accuracy in THA-AID and 99.7% held-out accuracy in TKA-AID—while adding a mechanism to decline or flag unfamiliar inputs [19,28]. One implements a three-state rule in which an input similar to less than 5.0% of the training distribution on two independent criteria is marked for discard, an input discrepant on one criterion is returned for human review, and only an input consistent on both is trusted. The other reports its detector as a diagnostic test rather than as a feature, giving both a true positive rate of 0.933 against images independently marked as outliers and a false positive rate of approximately 6.0% on images that were not.

The consequence appears when such a system meets data from outside its development setting [19]. In the record that reports it, 33.0% of the external test set was marked as a definite outlier and only 9.9% was classified as an inlier; on the internal test set, the corresponding figures were roughly 2.0% and around 10.0%. The classification accuracy on that external set was 97.0%, which is the figure a reader would ordinarily take away. Both statements are true of the same evaluation. The accuracy describes performance on the cases the system recognized; the outlier figures describe how few of them there were.

This is the property a closed-set accuracy figure cannot supply, and it is why uncertainty and failure-mode handling are reported as a separate evidence profile column rather than folded into performance. A system that cannot decline reports a number for every input, including the ones it should not have been given. These deployment concerns can be translated into practical engineering controls and staged implementation requirements.

Table 4 summarizes an implementation-readiness ladder that defines the evidence expected at each stage of translation, from technical feasibility to governance and local audit.

Table 6 reinforces the central implementation message of this review: arthroplasty AI should be judged not by model performance alone but by whether the system anticipates failure, exposes uncertainty, supports clinician oversight, and improves measurable workflow or clinical endpoints.

Building on these controls, the evidence reviewed here can be organized into a staged, surgeon-supervised deployment pathway, in which a tool advances only after meeting the requirements of the preceding stage. (1) Task and accountability definition: the bounded clinical task is specified, together with the clinician responsible for the output and an explicit statement of what the model does and does not decide. (2) Technical validation before clinical exposure: external, temporal, or multicenter performance, calibration, comparison with a clinically simple baseline, and behavior on out-of-distribution inputs are demonstrated rather than assumed. (3) Silent integration: the tool runs in parallel with usual care without influencing decisions, so that local performance, calibration drift, and workflow fit can be confirmed on the deploying center’s own data. (4) Supervised clinical use: the output informs the surgeon’s decision without replacing it, every result is reviewable and can be overridden, and low-confidence or out-of-distribution outputs are escalated. (5) Prospective monitoring and audit: performance, calibration, failure modes, and clinical or workflow endpoints are tracked over time, with predefined triggers to retrain, restrict, or withdraw the tool.

This pathway is deliberately conservative. It distinguishes two claims that this literature supports at very different rates. A system that is technically promising and workflow-compatible performs well on retrospective data, produces an output a clinician can verify, and fits an existing process; what such systems share is not accuracy but auditability. A system that is ready for clinical deployment must in addition have been evaluated outside the setting in which it was built, report performance in a form that supports a decision at an operating point, provide a mechanism for handling inputs it cannot process, and show that it changed something measurable in a real clinical or operational setting without harm elsewhere. The applications identified in this review sit in the first category. Across all stages, accountability for the clinical decision stays with the surgeon, and the AI is treated as an instrument under supervision rather than as a decision-maker.

### 4.8. Reporting Completeness and Methodological Appraisal

Reporting guidance for prediction models has been available throughout the period covered by this review, and AI-specific extensions were published during it [236,237,238,239]. Two directly relevant assessments highlighted in this synthesis used different instruments and units of assessment. Broader orthopedic reviews independently reported development-only designs, limited availability of external validation, partial TRIPOD completeness, and high risk of bias, but were retained as adjacent methodological corroboration rather than used to calculate frequencies in the included arthroplasty evidence [240,241,242].

Singh et al. assessed abstract compliance against four TRIPOD + AI criteria across two 18-month periods [243]. No abstract satisfied all four criteria. Performance measure specification was near-universal, whereas performance estimates with confidence intervals remained below one in five and did not increase; participant number reporting decreased. Shanmugaraj et al. appraised 24 shoulder arthroplasty prediction studies against the 22-item TRIPOD statement and PROBAST [110]. Mean adherence was 11.6 items, and two-thirds of studies were rated at high risk of bias. The two measurements use different instruments, units, periods, and samples and are not combined. A later systematic quality assurance review of 137 THA/TKA machine-learning studies similarly found high average reporting completeness alongside frequent high PROBAST risk, reinforcing that reporting adherence and risk of bias are related but non-interchangeable [244].

Incomplete reporting also constrains synthesis. One systematic review planned a meta-analysis and abandoned it because included studies did not report confidence intervals around discrimination estimates [34]. Another reconstructed intervals from reported standard errors where possible and used a multivariate random-effects model to account for multiple models within single studies [245]. These are downstream consequences of incomplete interval and model-level reporting, not merely editorial shortcomings.

Instruments now exist for trial protocols [246], trial reports [247], early-stage clinical evaluation [239], prediction model reporting [236,237], medical imaging AI [238], diagnostic accuracy studies [248], risk-of-bias appraisal [249,250], and calibration [207,251]. Across the 172 core primary-AI records, confirmed-present lower bounds were 27 (15.7%) for calibration, 10 (5.8%) for Brier score, eight (4.7%) for decision curve/net-benefit analysis, five (2.9%) for uncertainty/OOD handling, and eight (4.7%) for observed workflow or patient benefit. These values do not imply that all remaining records definitively omitted the item. Reporting completeness, risk of bias, applicability, calibration, and transport remain separate constructs.

### 4.9. Implications for Future Research

The next phase of arthroplasty AI should move beyond retrospective benchmark studies. The field needs fewer isolated models and more implementation-aware engineering. Several priorities follow from this review.

First, independent geographic, temporal, and site-separated evaluation should become routine, especially for prediction models, implant-recognition systems, imaging tools, NLP pipelines, and PJI-support models; local adaptation and frozen-model transport should be reported separately. Second, calibration and decision curve analysis should be reported for clinical prediction tasks, because discrimination alone does not define clinical usefulness. Third, studies should compare AI models with simple and interpretable clinical baselines, not just with other machine-learning algorithms. Fourth, uncertainty quantification and failure-mode reporting should be standard in imaging, implant recognition, and LLM studies. Fifth, prospective workflow-impact studies should evaluate whether AI changes decisions, reduces workload, improves planning, prevents errors, or improves patient experience.

The most promising future direction is multimodal arthroplasty AI. The building blocks already exist: radiographic models, implant classifiers, tabular prediction tools, operative-note NLP, radiology-report NLP, PJI biomarker models, PROM prediction, and LLM-based communication tools [17,18,19,20,21,25,26,27,77,78,79,80,81,82,146,147,148,149,152,153,154,157,158,188,205,208,226,227,228]. The translational opportunity is to integrate these components into accountable systems that combine imaging, implants, operative documentation, laboratory data, registry variables, and patient-reported outcomes. Such systems should be engineered around surgeon oversight, not autonomous replacement.

Although most arthroplasty AI studies remain retrospective, recent prospective cohort modeling after TKA indicates that the field is beginning to move toward prospective validation designs, which should become a routine expectation for clinically consequential models [252].

Future arthroplasty AI studies should report a minimum set of implementation-relevant items: the exact clinical task, intended user, input data source, reference standard, dataset split, evaluation setting, temporal separation, model adaptation status, calibration where applicable, comparison with simple clinical or statistical baselines, explainability or uncertainty approach, failure modes, missing data handling, dataset shift concerns, and the workflow action expected from the model output. For deployment-facing tools, prospective evaluation should include time saved, errors prevented, decision changes, workload reduction, patient understanding measures, or operational efficiency endpoints.

### 4.10. Strengths and Limitations

This review provides a broad and detailed structured synthesis of the literature on artificial intelligence in arthroplasty. Its breadth derives from a 252-publication evidence map spanning eight task families across hip, knee, shoulder, and less frequently examined arthroplasty settings, while its depth derives from publication-level assessment of validation design, model state at evaluation, uncertainty reporting, and workflow integration, with the principal evidence map counts traceable to publication-level supplementary records.

This review integrates clinical prediction, imaging automation, implant recognition, planning, NLP, PJI support, language model communication, robotics-linked workflows, and operational optimization across multiple arthroplasty domains. The source-level architecture, explicit applicability denominators, separate externality axes, lineage sensitivity, and detailed Supplementary Materials improve traceability and permit readers to inspect the basis of the synthesis.

Several limitations remain. The article is a structured critical narrative review rather than a prospectively registered systematic or scoping review, and its quantitative findings describe the included evidence set rather than field-wide prevalence. Literature selection, structured extraction, classification, and synthesis were performed by one author; standardized definitions and publication-level supplementary records improve transparency but do not provide independent duplicate assessment. Of 252 included publications, 247 were reviewed from full-text or authoritative source files, and five were retained as abstract-only contextual records; none contributed to primary quantitative results. Reviews and methodological publications were retained for context but were not counted as empirical model evaluations. Substantial heterogeneity in task, joint, input data, reference standard, outcome, evaluation design, and performance metric precluded meta-analysis. Rapidly changing areas such as language models, robotics-linked planning, and multimodal infection support may also evolve faster than the publication cycle.

These boundaries define the appropriate interpretation of the findings. The article provides a transparent critical synthesis of validation and implementation patterns within the included literature; it is not a pooled estimate of model performance or a formal certainty-of-evidence assessment.

## 5. Conclusions

Artificial intelligence in arthroplasty is best understood as supervised clinical engineering infrastructure for measurement, planning, documentation, registry construction, communication, and workflow. Two claims about that infrastructure need to be kept apart, because this literature supports one of them far more often than the other. Technically promising and workflow-compatible describes a system that performs well on retrospective data, produces an output a clinician can verify, and fits a process that already exists: implant recognition, automated radiographic measurement, component sizing and templating support, operative-note abstraction and registry-ready data extraction, and operative-time and scheduling models. What they share is not accuracy but auditability. Ready for clinical deployment is a different claim. It requires evaluation outside the setting in which the system was built, performance reported in a form that supports a decision at an operating point rather than a discrimination statistic, a mechanism for handling inputs the system cannot process, and evidence that the system changed something measurable in a real clinical or operational setting without harm elsewhere.

Applying that standard requires the evaluation design to be interpreted from the methods rather than from terminology alone. Within the included literature, similar labels were used for random internal partitions, multicenter development sets, later same-site cohorts, redevelopment on independent data, site-held-out evaluation, and geographically independent application of an unchanged model. Under the operational framework used here, these designs were assigned to separate categories because they answer different questions. This distinction is intended to improve evidence synthesis, not to criticize the original studies; in most cases, the underlying methods were sufficiently clear for the design to be classified transparently.

Broader models for readmission, satisfaction, PROM improvement, revision, chronic pain, and long-term outcomes remain clinically important but less consistently mature. Their value depends not on predictive discrimination alone but on calibration, external validation, interpretability, decision utility, and prospective workflow impact. Patient-facing large language models may improve education, consent support, and multilingual communication when supervised, but they should not be deployed as autonomous clinical authorities.

The central translational message is therefore one of engineered augmentation. Arthroplasty AI should integrate imaging, implants, and structured clinical data, quantify its own uncertainty, and stay accountable to the surgeon. Its most credible future is a human-in-the-loop system that strengthens surgical planning, documentation, infection assessment, patient communication, registry infrastructure, and operational efficiency while preserving surgeon responsibility—not an algorithm that replaces the surgeon.

## Figures and Tables

**Table 1:** Analytic framework for the structured critical narrative review.

Framework Domain	Operational Definition in This Review	Included Examples	Interpretation Rule
Population and clinical context	Patients undergoing, being evaluated for, or being followed after arthroplasty; arthroplasty-related imaging, operative documentation, registries, implant datasets, and perioperative workflows	THA, TKA, TSA, reverse TSA, TAA, TEA, thumb CMC arthroplasty, mixed TJA cohorts, arthroplasty imaging archives, operative notes, registry datasets	TMJ studies were treated only as adjacent joint reconstruction AI evidence and not as core arthroplasty evidence
AI concept	Use of AI or AI-adjacent computational methods in arthroplasty care, research, communication, planning, or workflow	ML, DL, computer vision, NLP, LLMs, chatbots, radiomics-based ML, AI-assisted templating, segmentation, optimization, finite-element/ML implant design	Conventional regression-only papers were excluded unless explicitly embedded in an AI/ML comparative or hybrid modeling framework
Clinical engineering task	The primary problem the AI system attempted to solve	Risk prediction, discharge prediction, PROM prediction, implant identification, radiographic measurement, templating, sizing, PJI support, NLP abstraction, patient education, robotics-linked planning, workflow optimization	Each source was assigned one dominant task for mapping; secondary roles were considered during synthesis
Data modality	Main input data used by the model or computational method	Structured clinical data, administrative data, registry data, PROMs, labs, radiographs, CT, MRI, operative notes, radiology reports, chatbot prompts, social media text, multimodal datasets	Data sources not clearly linked to the model task were not used for primary classification
AI family	Broad computational method used	Supervised ML, unsupervised ML, deep learning, computer vision, NLP, LLM/chatbot systems, radiomics, hybrid/multimodal AI, optimization	Algorithm-level details were retained when relevant but synthesized at the family/task level
Evidence type	Type of literature contributing to synthesis	Original studies, validation studies, registry/data-infrastructure studies, systematic reviews, scoping reviews, structured narrative reviews, methodological critiques, patient-facing AI evaluations	Review articles contextualized domain trends; original studies characterized empirical model development and validation signals
Validation maturity	Practical assessment of how model performance or clinical utility was evaluated	External validation, multicenter validation, temporal validation, internal validation, cross-validation, pilot evaluation, expert qualitative assessment for LLMs	Used for descriptive synthesis, not formal risk-of-bias scoring
Translational relevance	Degree to which the AI application could plausibly enter clinical workflow	PACS integration, registry extraction, revision planning, operating room scheduling, robotic planning, patient communication, surgeon-facing dashboards	Workflow relevance was considered separately from model performance metrics
Deployment concerns	Limitations relevant to safe clinical implementation	Dataset shift, calibration gaps, unclear failure modes, hallucination risk, unfamiliar implant classes, missing external validation, poor explainability	Used to identify engineering controls and future research priorities

Note: This framework was used to organize the included evidence set for structured critical narrative synthesis. It was not used as a formal risk-of-bias tool or evidence-certainty grading system. Using this framework, retained sources were organized at the reference level and then synthesized according to task, modality, validation maturity, and translational relevance.

**Table 2:** Operational taxonomy of validation, portability and redevelopment used in this review.

Operational Category	Operational Definition	Terms Authors Commonly Use for It	Counts Toward the Strict Geographic Numerator	Sensitivity Analysis Treatment
Internal random holdout	A partition drawn at random from one pooled collection, with no separation by site, time, or acquisition setting. Stratification that preserves the training distribution in the held-out part is a property of this category, not evidence against it.	Test set; held-out set; *external testing set*	No	Base case
Internal cross-validation	Repeated partitioning within one pooled collection. Resampling into mutually exclusive folds; repeated random splits into fixed proportions are recorded here and noted as not fold-exclusive.	Cross-validation; *k*-fold validation	No	Base case
Same-site temporal evaluation	Evaluation on a non-overlapping later period at the institutions that supplied development data.	Temporal validation; *external validation*	No	Reported separately; excluded from the strict numerator by rule
Prospective same-site evaluation	Evaluation on data accrued after the model was fixed, at the development site, with the model frozen before accrual began.	Prospective validation	No	Reported separately
Multicenter or multi-source development	Development data drawn from more than one institution or source, pooled before partitioning. A property of the development data, not of the evaluation.	*Multicenter study*; *multicenter validation*	No	Reported as a separate column; never merged with multicenter validation
Site-held-out evaluation within one organization	One or more sites withheld from development and used for evaluation, where all sites belong to the same organization, health system or data environment.	External validation; multicenter external validation	No	Reported in its own category; included only in a separately labeled broad site-separated sensitivity analysis
Independent geographic external evaluation	Evaluation at an organization that contributed no development data and does not share the development data environment, with the fitted model applied unchanged.	External validation	Yes	Base case
External portability with documented local adaptation	The model is applied at a site that contributed no development data, but is modified there before the reported evaluation. Out-of-box and post-adaptation performance are recorded separately where the study reports both.	External validation; multicenter validation; portability	No	Reported in the portability column; only a frozen pre-adaptation evaluation, where one exists, may enter the strict numerator
Local recalibration or updating	Model structure retained, coefficients or calibration re-estimated on data from the receiving setting.	External validation; model updating	No	Reported separately from transport
Independent data redevelopment	The model is refitted on the new data, including hyperparameter selection. A new model is evaluated on the data that produced it.	*External validation of previously developed algorithms*	No	Recorded as development, not as evaluation
Unresolved	The methods do not establish which of the above applies, and the reported description does not settle it. Recorded as unresolved rather than assigned to the most likely category.	—	No	Retained as unresolved in design-specific summaries; never converted to absence

Italicized terms in the third column are examples observed in the included literature for the design category shown in that row.

**Table 3:** Clinical engineering layers, translational signals, and deployment barriers in arthroplasty AI.

Clinical Engineering Layer	Representative AI Tasks	Current Bounded-Task or Workflow-Relevance Signal	Main Deployment Barrier	Near-Term Role
Clinical prediction and utilization modeling	Complication, mortality, readmission, discharge, LOS, PROM improvement, satisfaction, revision, transfusion, chronic pain	Useful for short-term triage and workflow-linked endpoints	Variable external validation, calibration gaps, modest gains over simple baselines	Surgeon-supervised counseling, perioperative triage, pathway selection
Imaging automation and implant recognition	Implant identification, cup-angle measurement, stem subsidence quantification, loosening detection, shoulder radiographic classification	Clearer bounded-task validation and human-verification pathway	Dataset shift, unfamiliar implants, image-quality variation, incomplete uncertainty reporting	Revision planning, radiographic surveillance, PACS/registry support
Templating, sizing, and planning	TKA component sizing, THA three-dimensional planning, implant-position prediction, radiomics-based press-fit prediction	Strong within-one-size sizing and growing three-dimensional planning evidence	Exact-size variability, implant-system dependence, limited multicenter testing	Preoperative planning, inventory preparation, reconstruction support
Robotics-linked segmentation and precision surgery	CT segmentation, alignment analytics, robotic-assisted planning, surgical-variable outcome modeling	Promising integration with procedural workflows	Platform specificity, limited prospective evaluation, unclear outcome impact	Enabling layer for image-guided and robotic arthroplasty
NLP and registry infrastructure	Operative-note abstraction, implant model extraction, registry variable extraction, fracture-event detection, radiology-report modeling	High local accuracy; external accuracy reported after site-specific refinement	Local documentation variation, scanned-document quality, need for harmonized data standards	Registry construction, quality improvement, multimodal AI infrastructure
PJI decision support	PJI diagnosis, recurrence prediction, biomarker probability scoring, infection-risk modeling, mortality stratification	High clinical relevance and multimodal data richness	Evolving definitions, culture-negative cases, label heterogeneity, limited prospective validation	Diagnostic support, risk stratification, revision planning
LLMs and patient communication	FAQ answering, consent support, postoperative counseling, multilingual engagement, chatbot follow-up	Rapidly expanding and potentially scalable	Hallucination, incompleteness, medicolegal uncertainty, lack of personalization	Supervised patient education and communication support
Workflow and operations engineering	Operating room scheduling, operative-time prediction, resource allocation, outpatient pathway selection	Operational endpoints can be more meaningful than AUC	Trade-offs between overtime, underutilization, throughput, staffing, and patient flow	System-level efficiency support and implementation research
Underrepresented arthroplasties	Ankle, elbow, and thumb CMC arthroplasty; adjacent TMJ imaging considered only contextually	Ankle has the most developed evidence among underrepresented arthroplasties	Small datasets, sparse validation, limited procedure-specific infrastructure	Early-stage domain-specific model development

Note: The table summarizes the final included evidence by functional layer and translational role. It is not a pooled effect estimate, formal evidence-certainty rating, or ranking of clinical readiness. Detailed publication-level data are provided in Supplementary Table S1A,B.

**Table 4:** Implementation-readiness ladder for arthroplasty AI.

Level	Implementation-Readiness Stage	Minimum Evidence Needed	Arthroplasty Example and Deployment Implication
1	Technical feasibility	Model performs a defined task in a development dataset with a clear input, output, and reference target.	Example: Radiograph classifier, sizing model, or PROM predictor developed on local data. Feasibility alone does not justify clinical use.
2	Internal validation	Held-out testing, cross-validation, bootstrapping, or temporal split within the development environment.	Example: Internal AUC, accuracy, Dice, MAE, F1, or calibration results. Risk: performance may reflect site-specific documentation, implant mix, or imaging protocol.
3	Independent geographic, temporal, or site-separated evaluation	Transportability tested across institution, time period, scanner/PACS setting, implant library, registry, or population.	Example: Implant-identification or NLP model tested outside the source environment. Risk: dataset shift, rare implant classes, or documentation-style drift.
4	Comparator, calibration, and decision utility	Comparison with human experts, manual measurements, operative records, registry labels, simple clinical baselines, calibration plots, Brier score, or decision curve analysis.	Example: PROM prediction compared with preoperative PROM thresholds; component measurement compared with expert measurement. Risk: model may not add value beyond simpler tools.
5	Explainability, uncertainty, and failure-mode handling	Feature attribution, saliency maps, conformal prediction, confidence intervals, outlier detection, image-quality gates, or explicit failure-mode analysis.	Example: Implant-recognition system with conformal prediction and out-of-distribution detection. Risk: overconfident erroneous output or automation bias.
6	Workflow integration	Tool tested in the environment where action occurs: PACS, registry, EHR, operating room scheduling, patient portal, robotic planning platform, or revision-preparation workflow.	Example: Operative-note NLP feeding a registry, or operating room duration prediction supporting scheduling. Risk: accurate output may still fail to change workflow.
7	Prospective clinical or operational impact	Prospective evaluation of decision change, time saved, error reduction, workload reduction, patient understanding, cost, safety, or outcome improvement.	Example: Randomized or prospective AI-assisted planning, consent, or scheduling evaluation. Risk: retrospective accuracy may not translate into clinical benefit.
8	Governance, monitoring, and local audit	Defined accountability, user override tracking, drift monitoring, version control, recalibration plan, local quality assurance, and escalation pathways.	Example: Institutional deployment plan for LLM communication, PJI diagnostic support, or imaging AI. Risk: silent performance decay, medicolegal ambiguity, and unsafe scaling.

Note: The ladder is an implementation-readiness guide, not a formal regulatory classification or evidence-certainty scale. A higher level requires the evidence from preceding levels to remain valid in the intended clinical environment.

**Table 5:** Illustrative validation design patterns and their interpretation under the operational framework.

Evaluation Pattern	Terminology Reported in Representative Publications	Classification Used in This Review	Interpretation for Evidence Synthesis	Representative Publications
Random partition of one pooled multi-institutional image collection	External testing dataset	Internal random holdout	Diversity in the pooled development collection does not establish site-independent evaluation	Karnuta 2021 hip and knee [17,87]
Multicenter development data followed by one pooled random split	Multicenter study	Multicenter or multi-source development	Multicenter development and multicenter external evaluation are separate properties	Kunze 2023 [97]
Later non-overlapping cohort from the same institution	External validation	Same-site temporal evaluation	Provides temporal evidence but not geographic/site independence	Wongsak 2025 [124]
New data source with renewed feature selection and model fitting	External validation of previously developed algorithms	Independent data redevelopment	Evaluates a newly fitted model rather than transport of the original fitted artifact	Buddhiraju 2023 [206]
Site-held-out evaluation within the same health organization	External validation	Site-held-out evaluation within one organization	Stronger than a random split, but distinct from evaluation outside the shared organization/data environment	Farrow 2024 and ARCHERY protocol [205,213]
Institutions withheld from development, with the fitted model applied unchanged	Multicenter external validation	Strict frozen-model geographic external evaluation	Supports evaluation at institutions that did not contribute development data	Karnuta 2023 hip and knee [214,215]

Note: The table illustrates how the operational definitions distinguish design patterns. It does not assign fault to individual publications, and it does not imply that author terminology was unreasonable within the context of the original report. Member-level classifications, applicability routes, and terminology-mapping flags are provided in Supplementary Table S1B. Detailed source locators were used during assessment but are not included in the public Supplementary Materials.

**Table 6:** Failure modes and engineering controls for arthroplasty AI.

AI Domain	Main Failure Mode	Why It Matters Clinically	Engineering Control	Deployment Endpoint
Implant recognition	Unknown implant, low-quality radiograph, unfamiliar projection, out-of-distribution implant class	Wrong implant identification may compromise revision planning and inventory preparation	Uncertainty quantification, outlier detection, image-quality gates, implant library coverage reporting	Correct implant identification, reduced revision-planning time, fewer inventory mismatches
Radiographic measurement	Segmentation error, projection variability, poor landmark detection	Inaccurate component-position or migration estimates may mislead surveillance	Human-verifiable measurements, confidence intervals, repeatability testing, external validation	Measurement accuracy, interobserver reduction, surveillance efficiency
Templating and sizing	Exact-size error, implant-system dependence, population shift	Poor planning may affect inventory, operative efficiency, and reconstruction fidelity	Within-one-size reporting, implant-system validation, multicenter testing, surgeon override	Planning accuracy, inventory efficiency, operative preparedness
Clinical prediction	Poor calibration, weak external validation, modest benefit over simple rules	Risk estimates may mislead counseling or pathway selection	Calibration plots, decision curve analysis, external validation, baseline comparator models	Improved counseling, pathway selection, reduced avoidable complications or utilization
PROM and satisfaction prediction	Outcome confounding, expectation effects, baseline PROM dominance	Model may overpromise individualized outcome prediction	Expectation-sensitive variables, interpretable models, prospective validation	Better shared decision-making and expectation alignment
NLP abstraction	Documentation variation, scanned-document errors, entity misclassification	Registry variables may be incomplete or wrong	Local validation, human audit, harmonized data dictionaries, error monitoring	Registry completeness, abstraction accuracy, reduced manual chart review
PJI support	Label heterogeneity, evolving definitions, culture-negative cases	Misclassification can alter revision strategy, antibiotics, and prognosis	Standardized PJI criteria, multimodal validation, prospective testing, expert review	Diagnostic support, recurrence-risk stratification, revision planning
LLM patient communication	Hallucination, incomplete advice, overconfident language, poor personalization	Patients may misunderstand risks, recovery, or indications	Retrieval augmentation, clinician-approved content, escalation triggers, readability targets	Safer education, reduced anxiety, improved access, better preparation for consultation
Robotics-linked planning	Platform specificity, segmentation failure, unclear outcome effect	Technical accuracy may not translate into better surgery or outcomes	Platform-specific validation, operative endpoint reporting, surgeon-in-loop verification	Segmentation accuracy, operative efficiency, alignment or reconstruction quality
Workflow optimization	Hidden trade-offs between overtime, underutilization, throughput, and staffing	A favorable metric may worsen another operational outcome	Multi-objective optimization, transparent trade-off reporting, local simulation	Operating room utilization, overtime, throughput, staff burden, patient flow

Note: The proposed controls are intended as translational design principles for clinician-supervised arthroplasty AI. They are not regulatory requirements or formal risk-of-bias criteria.

## Data Availability

No primary patient-level datasets were generated or analyzed for this article. All public materials supporting the structured critical review are included in the article and Supplementary Materials.

## Supplementary Materials

The following supporting information can be downloaded at: https://www.mdpi.com/article/10.3390/bioengineering13091036/s1. Supplementary Table S1A: Reference map of the 252 included publications; Supplementary Table S1B: Publication-level evidence-map fields; Supplementary Table S2: Literature-identification, source-verification, and analytical-applicability framework; Supplementary Table S3: Descriptive evidence-profile and transportability rubric; Supplementary Table S4: Criterion-specific evidence profile by dominant task. References [1,2,3,4,5,6,7,8,9,10,11,12,13,14,15,16,17,18,19,20,21,22,23,24,25,26,27,28,29,30,31,32,33,34,35,36,37,38,39,40,41,42,43,44,45,46,47,48,49,50,51,52,53,54,55,56,57,58,59,60,61,62,63,64,65,66,67,68,69,70,71,72,73,74,75,76,77,78,79,80,81,82,83,84,85,86,87,88,89,90,91,92,93,94,95,96,97,98,99,100,101,102,103,104,105,106,107,108,109,110,111,112,113,114,115,116,117,118,119,120,121,122,123,124,125,126,127,128,129,130,131,132,133,134,135,136,137,138,139,140,141,142,143,144,145,146,147,148,149,150,151,152,153,154,155,156,157,158,159,160,161,162,163,164,165,166,167,168,169,170,171,172,173,174,175,176,177,178,179,180,181,182,183,184,185,186,187,188,189,190,191,192,193,194,195,196,197,198,199,200,201,202,203,204,205,206,207,208,209,210,211,212,213,214,215,216,217,218,219,220,221,222,223,224,225,226,227,228,229,230,231,232,233,234,235,236,237,238,239,240,241,242,243,244,245,246,247,248,249,250,251,252] are cited in the Supplementary Materials.

## Abbreviations

ACS	American College of Surgeons
AI	artificial intelligence
AI-HIP	artificial intelligence-assisted hip-planning software
ARCHERY	Artificial intelligence to Revolutionise the patient Care pathway in Hip and knEe aR-throplastY
AUC	area under the curve
AUROC	area under the receiver operating characteristic curve
CE	Conformité Européenne
CMC	carpometacarpal
CNN	convolutional neural network
CT	computed tomography
DL	deep learning
DVT	deep vein thrombosis
ED	emergency department
EHR	electronic health record
F1	F1 score (harmonic mean of precision and recall)
FAQ	frequently asked question
HADS-A	Hospital Anxiety and Depression Scale–Anxiety subscale
ICC	intraclass correlation coefficient
ICM	International Consensus Meeting
LLM	large language model
LOS	length of stay
MAE	mean absolute error
ML	machine learning
MEDLINE	Medical Literature Analysis and Retrieval System Online
MRI	magnetic resonance imaging
NHS	National Health Service
NLP	natural language processing
OAI	Osteoarthritis Initiative
OOD	out-of-distribution
PACS	picture archiving and communication system
PCA	patient-controlled analgesia
PJI	periprosthetic joint infection
PRISMA-ScR	Preferred Reporting Items for Systematic Reviews and Meta-Analyses extension for Scoping Reviews
PROBAST	Prediction model Risk of Bias ASsessment Tool
PROM	patient-reported outcome measure
PSI	patient-specific instrumentation
QALY	quality-adjusted life-year
SF-36	36-Item Short Form Health Survey
TAA	total ankle arthroplasty
TEA	total elbow arthroplasty
THA	total hip arthroplasty
TJA	total joint arthroplasty
TKA	total knee arthroplasty
TMJ	temporomandibular joint
TRIPOD	Transparent Reporting of a multivariable prediction model for Individual Prognosis Or Diagnosis
TSA	total shoulder arthroplasty
WOMAC	Western Ontario and McMaster Universities Osteoarthritis Index
