# Supplementary material for: Artificial Intelligence in Arthroplasty: A Comprehensive Structured Critical Review and Descriptive Evidence Map of Validation, Uncertainty, and Workflow Integration
Source: Bioengineering (Basel). 2026 Sep 6;13(9):1036. doi: 10.3390/bioengineering13091036 (PMC13603719; doi:10.3390/bioengineering13091036)
Supplement: Supplementary file 1 [file bioengineering-13-01036-s001.zip › Supplementary Table S2 31.08.2026.pdf]

Supplementary Table S2. Literature-identification, source-verification, and analytical-applicability framework

This table summarizes the literature-identification process, source-verification depth, and denominators used for separate analytical questions in the structured critical narrative review. It is not presented as a PRISMA systematic-review flow.

Component	Final value	Status	Interpretation
Literature-identification period	1 January–17 August 2026	Final	Literature identification (database searching, citation tracking, and manual record retrieval), source acquisition, and source verification were conducted within this period.
Search sources	PubMed/MEDLINE; Web of Science Core Collection; Scopus; Google Scholar; backward and forward citation checking	Final	Google Scholar supported identification of difficult-to-locate publications and citation checking rather than functioning as a fully reproducible Boolean database.
Included evidence set	252 publications	Final	All included publications are represented in the manuscript and Supplementary Table S1A.
Source depth	247 full-text/authoritative-source reads + 5 abstract-only records	Final	None of the five abstract-only records contributes to a primary quantitative result.
Task-distribution framework	172 core arthroplasty-specific primary AI publications	Final	Denominator for T1–T8 task mapping in Figure 2.
Transportability framework	150 publications with an assessable fitted model or operational system	Final	Denominator for transportability percentages; publications outside this set were not counted as negative external validations.
Overlap of analytical sets	139 publications	Final	The 172- and 150-publication sets overlap but answer different questions.
Applicability routing	150 transport-assessable / 5 measurement-validation / 92 not applicable / 5 unresolved	Final	The routing categories describe applicability to the transportability question, not inclusion or exclusion from the review; publication-level routes are provided in Supplementary Table S1B.
Manuscript representation	252 numbered citations	Final	References are numbered 1–252 in first-appearance order. Publication-level applicability routes are provided in Supplementary Table S1B.
