# Supplementary material for: Artificial Intelligence in Arthroplasty: A Comprehensive Structured Critical Review and Descriptive Evidence Map of Validation, Uncertainty, and Workflow Integration
Source: Bioengineering (Basel). 2026 Sep 6;13(9):1036. doi: 10.3390/bioengineering13091036 (PMC13603719; doi:10.3390/bioengineering13091036)
Supplement: Supplementary file 1 [file bioengineering-13-01036-s001.zip › Supplementary Table S3 31.08.2026.pdf]

Supplementary Table S3. Descriptive evidence-profile and transportability rubric

This rubric is descriptive and is not a formal risk-of-bias instrument, evidence-certainty grade, regulatory-readiness assessment, or comparative-effectiveness ranking. The underlying n/N values are the primary results; the bands are descriptive visual aids only. No evidence-set-wide T0–T4 maturity prevalence was assigned.

Criterion	Eligibility	Positive signal	Not positive	Output
Geographic/site-separated evaluation	Transport-assessable core primary-AI publication	Distinct institution/site; model state assessed separately	Internal split, same environment, or insufficient information	n/N + band
Later non-overlapping temporal evaluation	Transport-assessable core primary-AI publication	Evaluation later and non-overlapping with development	Different dates alone, overlap, or insufficient information	n/N + band
Multicenter/multi-source development	Core primary-AI publication	Confirmed development data from multiple institutions or sources	Multicenter label without confirmed multi-source development	Confirmed lower-bound n/N + band
Performance completeness/decision utility	Eligible probabilistic prediction/diagnostic publications	Calibration or overall-error evidence together with a class-sensitive or decision-utility signal	Discrimination alone or nonapplicable task	n/N + band
Uncertainty/OOD handling	Core primary-AI publication	Abstention, OOD/outlier detection, conformal prediction, or explicit failure handling	Feature attribution alone	Confirmed lower-bound n/N + band
Observed workflow/patient benefit	Core primary-AI publication	Observed operational or patient-centered endpoint	Modeled/simulated benefit or technical accuracy alone	Confirmed lower-bound n/N + band

Band rules

Band	Rule	Interpretation
absent	0.0%	No confirmed signal among eligible publications.
sparse	>0.0% and <25.0%	Signal present in a minority.
emerging	≥25.0% and <50.0%	Signal visible but not recurrent.
recurrent	≥50.0%	Signal present in at least half.
no band	Eligible denominator <4	Report n/N only.

Transportability schema

Axis	Question	Final status
1 Geographic/site	Was evaluation performed in a distinct environment?	Complete for 150/150 applicable publications.
2 Temporal	Was evaluation later and non-overlapping?	Complete for 150/150 applicable publications.
3 Model state	Was the model applied unchanged or locally adapted?	Complete for 150/150 applicable publications.
4–8 Extended qualifiers	Investigator, funding/program, reference-standard, product/domain, and open-set independence	Retained as publication-level qualifiers; not populated across the full evidence set.
